# TSPAN32 as a biomarker associated with radiotherapy and immune microenvironment remodeling in lung adenocarcinoma

**DOI:** 10.3389/fonc.2026.1724489

**Published:** 2026-04-14

**Authors:** Guomin Huang, Haoyue Hu, Lanlan Guo, Mingying Xiao, Wei Chen, Yuanyang Huang, Xin Yang, Zhijie Li, Xuan Li, Ming Chen, Xiaoyan Huang

**Affiliations:** 1 State Key Laboratory of Oncology in South China, Guangdong Provincial Clinical Research Center for Cancer, Sun Yat-sen University Cancer Center, Guangzhou, Guangdong, China; 2 United Laboratory of Frontier Radiotherapy Technology of Sun Yat-sen University & Chinese Academy of Sciences Ion Medical Technology Co., Ltd, Guangzhou, Guangdong, China; 3 Precision Radiotherapy Research Center, Advanced Energy Science and Technology Guangdong Laboratory, Huizhou, China

**Keywords:** immune infiltration, lung adenocarcinoma, radiotherapy, tetraspanin 32, transcriptomics

## Abstract

**Introduction:**

Lung cancer is a high-mortality-rate malignant tumor. Radiotherapy combined with immunotherapy shows great potential in improving patient survival. This study aims to identify gene sets associated with radiotherapy effectiveness and immune infiltration in lung adenocarcinoma (LUAD), construct a prognostic model, and preliminarily evaluate the effectiveness of the key gene TSPAN32 as a predictive diagnostic marker and therapeutic target for LUAD.

**Methods:**

Genomic datasets from The Cancer Genome Atlas (TCGA) were analyzed to develop a 12-gene risk score model. The prognostic value of TSPAN32 was assessed, and its correlation with immune infiltration was examined. *In vitro* and *in vivo* experiments were conducted to validate the biological functions of TSPAN32 in LUAD cell lines A549 and H1299.

**Results:**

The 12-gene risk score model accurately predicts prognosis and correlates with immune infiltration. TSPAN32, with high weight in the model, can independently predict prognosis and is associated with immune infiltration. TSPAN32 is significantly downregulated in LUAD cells. Its overexpression significantly inhibits the proliferation and migration of A549 and H1299 cells, enhances radiosensitivity, and may exert biological effects through the TSPAN32-PTEN signaling axis.

**Discussion:**

These findings suggest that TSPAN32 could serve as a predictive biomarker for LUAD patients who may benefit from combined radiotherapy and immunotherapy, providing a theoretical basis for optimizing treatment strategies and improving patient prognosis.

## 1 Introduction

Lung cancer is the leading cause of cancer-related death worldwide, and more than one million new cases are diagnosed globally each year (1). Approximately 80%-85% of these cases are classified as non-small cell lung cancer (NSCLC). Lung adenocarcinoma (LUAD) accounts for 40%-50% of NSCLC cases. Because early symptoms are insidious, approximately 60% of patients present at an advanced or metastatic stage (stage IIIB-IV) at the time of diagnosis, and the five-year survival rate is less than 20% (2). Radiotherapy holds a pivotal position in the therapeutic strategy for LUAD, with epidemiological studies demonstrating that 30%-50% of patients receive radiation therapy at various disease stages (3). In medically inoperable early-stage patients, stereotactic body radiation therapy has been established as a definitive, curative-intent alternative (4), whereas in locally advanced disease (stage III) it confers significant survival benefit (5). Nevertheless, radiotherapy is associated with clinically significant limitations, including acute toxic effects such as radiation pneumonitis (incidence 10%-20%) and radiation-induced esophagitis. Moreover, its efficacy in controlling widespread metastatic lesions remains limited, necessitating synergistic integration with systemic therapies. The integration of immunotherapy into the clinical management of LUAD was initiated in 2015 with the regulatory approval of the inaugural PD-1 inhibitor, nivolumab, for advanced NSCLC, achieving an objective response rate of 19.4% (6). Emerging evidence also indicates that only 15%-20% of patients achieve sustained clinical responses (7), necessitating combination with radiotherapy or other modalities to potentiate therapeutic efficacy.

The confluence of radiotherapy and immunotherapy constitutes a seminal advance in the therapeutic landscape of lung cancer. This strategy is predicated upon the radiobiological phenomenon of the abscopal effect: beyond its localized cytotoxicity, ionizing radiation engenders a systemic activation of anti-tumor immunity that exerts anti-neoplastic effects on non-irradiated lesions (8). The advent of immune checkpoint inhibitors in 2015 marked a therapeutic paradigm shift, accelerating the development of multimodal treatment strategies. This was exemplified by the pivotal PACIFIC study, which established durvalumab as a consolidative therapy capable of significantly improving progression-free survival in unresectable stage III NSCLC patients (9). Contemporary clinical investigations have extended this paradigm to metastatic lung cancer; for instance, the phase II KEYNOTE-799 trial demonstrated that pembrolizumab administered concurrently with chemoradiotherapy achieved an objective response rate of 70% (10). However, this approach faces considerable hurdles such as overlapping toxicities and heterogeneous radiotherapy regimens. Most notably, the absence of reliable predictive biomarkers for patient stratification remains a fundamental limitation in radioimmunotherapy for lung cancer. Addressing this biomarker deficit has emerged as an imperative research priority to enable precision application of combined modality therapy.

In this study, we employed transcriptomic profiling coupled with least absolute shrinkage and selection operator (LASSO) COX regression analysis incorporating 100-fold cross-validation to identify radiation response-associated gene signatures in LUAD patients. Based on the results, a 12-gene risk score model was developed, and its predictive correlation with immunotherapy response was systematically investigated. Through comprehensive analyses, TSPAN32 (tetraspanin 32) was identified as a potential prognostic biomarker for LUAD patients receiving combined radiotherapy and immunotherapy. *TSPAN32* is located on human chromosome 11q24 and encodes a 247-amino-acid transmembrane protein with four transmembrane domains; the encoded protein, TSPAN32, belongs to the tetraspanin (Tspan) family. Tspan is ubiquitously expressed across all multicellular eukaryotes and exhibit a high degree of evolutionary conservation among species (11). A total of 34 Tspan proteins have been identified in mammals, of which 33 have been definitively characterized in humans (12). These proteins possess a conserved tetraspan membrane topology, consisting of a small extracellular loop, a large extracellular loop, and intracellular N- and C-termini. This configuration enables the sequestration of multiple partner proteins into discrete membrane microdomains termed Tspan-enriched microdomains, thereby orchestrating signal transduction (13). Accumulating evidence demonstrates that Tspan proteins orchestrate a spectrum of biological processes, including cell migration, adhesion, activation, and apoptosis (14, 15). Notably, TSPAN8 and TSPAN13 have been causally implicated in tumor metastasis and progression (16, 17), however, investigations focusing on TSPAN32 remain scarce.

Members of the Tspan family (Tspan1-Tspan33) can exert either tumor-promoting or tumor-suppressive effects in a cancer-type-dependent manner. According to analyses in the GEPIA2 database (http://gepia.cancer-pku.cn/), *TSPAN32* is significantly underexpressed in LUAD. Notably, study has demonstrated that its overexpression in leukemia effectively inhibits disease progression by impeding the proliferation of leukemia stem cells (18). Members of this protein family are intimately linked to the tumor immune microenvironment and modulate host immune responses through multiple mechanisms: (1) Regulation of antigen presentation: TSPAN29 associates with the MHC class II complex (19), whereas TSPAN30 suppresses antigen presentation (20). (2) Modulation of immune-cell trafficking: TSPAN26 and TSPAN28 have been shown to promote dendritic-cell migration, whereas TSPAN27 inhibits it (21). (3) Regulation of immune cell function: Deficiency of TSPAN28 impairs the suppressive capacity of regulatory T cell (Treg) and myeloid-derived suppressor cell (MDSC) populations (22), while TSPAN7 restrains immunosuppressive cells and participates in lymphocyte recruitment (23). (4) Modulation of immune checkpoints, TSPAN9 expression is negatively correlated with CTLA4 and influences immune-cell infiltration (24). (5) Participation in innate immune signaling: TSPAN27 modulates TLR9-mediated pathogen recognition (25). Furthermore, apart from TSPAN19, all other Tspan proteins are expressed in at least one B-lymphoma cell line (26). In summary, the Tspan family exerts multifaceted control over tumor cell biology and the tumor immune microenvironment, endowing it with substantial potential as both diagnostic biomarker and therapeutic target. In this study, we developed a prognostic model for radiotherapy efficacy in LUAD and, through integrative analyses of immunotherapy relevance, identified TSPAN32 as a pivotal molecule. Subsequent mechanistic investigations delineated its role in modulating radiosensitivity and remodeling the immune landscape, thereby offering novel theoretical insights into the molecular foundations of radio-immunotherapy for LUAD.

## 2 Materials and methods

### 2.1 Data collection and processing

RNA-seq data and microsatellite status information for 600 LUAD samples were downloaded from The Cancer Genome Atlas database (TCGA) (https://www.cancer.gov/tcga). We selected and analyzed 582 samples with protein-coding gene expression matrices, including 524 tumor samples and 58 healthy samples in the present study. The clinical data of 1,034 cases were obtained from the TCGA database. A total of 500 cases were retained after excluding samples with missing prognosis and survival status information. These clinical samples were subsequently matched with the corresponding transcriptomic data for further analysis. Compared to the control group, a total of 5,389 differentially expressed genes were identified in the tumor group.

A total of 126 radiotherapy-related gene files for LUAD were downloaded from the TCGA database. After excluding samples without recorded radiotherapy efficacy, 46 cases remained, including 16 cases in the remission group (complete or partial remission) and 30 cases in the non-remission group (stable disease or disease progression). A total of 152 genes were found to be differentially expressed when comparing the disease remission group and the non-remission group. The intersection of differentially expressed genes from the disease remission group after radiotherapy and the tumor group were further analyzed, resulting in the identification of 111 genes for subsequent analysis.

One validation cohort, GSE72094 were applied in the present study (https://www.ncbi.nlm.nih.gov/geo/). The dataset GSE72094 was annotated by the GPL15048 platform (27), which is a large cohort of resected tumors from 442 lung adenocarcinoma (LUAD) patients with data including annotation of prevalent driver mutations (KRAS and EGFR) and tumor suppressor mutations (STK11 and TP53), microarray-based gene expression and clinical covariates including overall survival (OS).

### 2.2 Identification of differentially expressed genes

The “DESeq2” package of R was applied to identify DEGs between normal and tumor samples, between disease response and non-response in LUAD patients after radiotherapy, and between high-risk and low-risk groups in the training cohort. |Log_2_ (fold change)| > 2 and adjusted p < 0.05 were the criteria for defining DEGs. DEGs were visualized using volcano plots generated with ggplot2 and heatmaps created with the pheatmap package in R. The differentially expressed genes common between the disease response group and the tumor group after radiotherapy were displayed using a Venn diagram.

### 2.3 Construction of the prognostic model

To identify marker genes for key cell populations, we constructed a training set from the intersection of genes differentially expressed in lung cancer and genes linked to radiotherapy response. The GSE72094 dataset served as the validation set. The prognostic model was constructed using LASSO regression. For each patient, a risk score was calculated by integrating the expression values of the signature genes, each weighted by its corresponding LASSO regression coefficient. The precision of the model predictions was assessed by a receiver operating characteristic (ROC) curve.

The radiotherapy-related genes were screened by univariate COX regression, LASSO regression analysis and multivariate COX regression analysis to construct a novel prognostic gene signature. Each sample’s risk score was calculated using the following formula:


Risk score = ∑ expgenei ∗ βi

where expgenei represents the expression level of the i-th gene, and βi represents the regression coefficient corresponding to the i-th gene. In all participating cohorts, the samples were divided into low- and high-risk groups based on the risk score (median cut-off value). To analyze the survival conditions for the prognosis signature, the optimized cut-off and the Kaplan-Meier survival curve were generated using R packages “survival” and “survminer”. The predictive performance was evaluated using ROC curve, risk plot, and concordance index (C-index). Detailed information for prognostic genes was obtained from National Center for Biotechnology Information (NCBI, https://www.ncbi.nlm.nih.gov/).

### 2.4 Construction and valuation of nomogram

Risk score and clinical factors including gender, race, vital status, age, T stage, N stage, M stage, tumor stage, survival time, and death status were analyzed using univariate COX regression analysis to screen the factors significantly related to survival. Then, multivariate COX regression analysis was applied to identify the candidate predictors significantly related to survival (p < 0.05). Finally, the patients’ survival outcomes in 1-, 2-, 3- and 4-year can be calculated using the total score and the probability of survival. The discrimination and accuracy of the nomogram model were assessed using ROC curves, calibration curves, and decision curve analysis.

### 2.5 Immunoinfiltration analysis

Various bioinformatics methods were employed to analyze the characteristics of immune cell infiltration in the tumor microenvironment (TME) and to evaluate their correlation with prognosis indicators. Data processing was conducted using the R packages “tidyverse” and “IOBR”. Immune cell infiltration analysis was performed using the CIBERSORT algorithm, a linear regression-based method that infers the relative proportions of different immune cell types in the TME from RNA-seq data. The permutation number was set to 100 to preliminarily assess the significance of the results. The immune score was calculated using the ESTIMATE algorithm implemented in the R package “estimate”, and its correlation with risk scores was evaluated. Additionally, Tumor Immune Dysfunction and Exclusion (TIDE) analysis was conducted using the TIDE tool to assess immune dysfunction and exclusion in tumors. Based on pre-treatment tumor expression profiles, TIDE can predict patient responses to immunotherapy.

To further explore these relationships, clinical data from The Cancer Imaging Archive (TCIA) were integrated with TCGA data to investigate the associations between immune cell infiltration and clinical indicators, such as the expression levels of immune checkpoints. Finally, the immune characteristics of LUAD were analyzed using the IMvigor210 dataset. Patients were stratified into high-risk and low-risk groups based on calculated risk scores, and survival analysis was performed.

### 2.6 GO, KEGG, GSEA and GSVA analyses

Gene Ontology (GO) and Kyoto Encyclopedia of Genes and Genomes (KEGG) enrichment analyses were performed using the R packages “clusterProfiler”, “org.Hs.eg.db”, and “tidyverse” to investigate the biological functions of the 12 differentially expressed genes. Subsequently, to explore potential synergistic or gene-specific biological roles, an independent analysis was conducted on the remaining 11 genes after excluding *TSPAN32*.

Patients were categorized into high and low gene expression groups based on gene expression profiles. To further explore the differences in signaling pathways between these two groups, Gene Set Enrichment Analysis (GSEA) was utilized. The background gene set, specifically annotated for subtype pathways, was sourced from the Molecular Signatures Database (MSigDB). Differential expression analysis was then performed to compare pathways across subtypes. Significantly enriched gene sets were identified using consistency scores, with an adjusted p < 0.05.

Gene Set Variation Analysis (GSVA) is an unsupervised method that converts gene-level expression data into pathway-level activity scores, enabling the assessment of the enrichment of specific biological functions or pathways in individual samples. In this study, gene sets were obtained from the Molecular Signatures Database (version 7.0), and the GSVA algorithm was employed to precisely calculate the enrichment scores for each gene set. This analytical strategy facilitated the evaluation of potential differences in biological state across diverse samples.

### 2.7 Cell culture

The normal lung cell BEAS-2B, LUAD cell lines A549 and H1299, and the Human Embryonic Kidney 293T cells (HEK-293T) were obtained from American Type Culture Collection (ATCC). Cells were cultured in DMEM (HEK-293T) or RPMI 1640 medium (Gibco, Thermo Fisher Scientific) supplemented with 10% fetal bovine serum (FBS, HyClone). Cells were routinely cultured in a humidified atmosphere containing 5% CO_2_ at 37 °C.

### 2.8 Irradiation parameters

In this study, irradiation was performed using an RS-2000 Pro 225 biological X-ray irradiator (Rad Source Technologies, USA). The device is equipped with a tungsten-target fixed-anode X-ray tube (maximum tube voltage 225 kV, maximum tube current 17.7 mA, maximum power 4.2 kW, 0.3 mm copper alloy filter), utilizing X-ray as an alternative to traditional radioisotope sources. The irradiation parameters were set as follows: dose rate 1 Gy/min, source-to-surface distance 50 cm, field diameter 20 cm, equipped with a boron carbide RAD+ reflector, and dose uniformity ≥95%. The device is calibrated annually by the manufacturer using the accompanying ionization chamber, and all irradiation procedures in this study were performed within the validity period of calibration.

### 2.9 siRNA, plasmid, and lentivirus

The siRNA targeting *TSPAN32* (sequence information shown in **Supplementary Table 8**) and the TSPAN32 overexpression plasmid were purchased from Genepharma (Shanghai, China). Cells were seeded into 6-well plates and transfected when they reached approximately 60% confluence. For siRNA transfection: 75 pmol of siRNA per well was diluted in 42.5 μl Buffer and transfected using GP-transfect-mate transfection reagent (GenePharma). For plasmid transfection: 3 μg of overexpression plasmid per well was transfected using 3.4 μl Lipofectamine 3000 (Thermo Fisher Scientific, L3000015). The shRNA targeting TSPAN32 was purchased from Genepharma (Shanghai, China). Lentiviral packaging was performed using HEK-293T cells. For HEK-293T cells in a 10 cm culture dish, 15 µg of target plasmid, 6 µg of psPAX2 (Addgene), 2.4 µg of pMD2.G (Addgene) were mixed with 24 µl linear polyethyleneimine (Yisheng). Viral supernatants were collected at 24, 48, and 72 h post-transfection and used to infect LUAD cells (A549).

### 2.10 Biological effect assessment

A549 and H1299 cells were subjected to transfection alone or in combination with 4 Gy X-ray irradiation. Subsequent biological assessments were conducted as follows: for cell proliferation analysis, cells were seeded in 96-well plates at a density of 5,000 cells per well and measured by MTT assay at 0, 24, 48, and 72 h post-transfection (MTT reagent: Beyotime, ST1537);cell migratory capacity was evaluated by wound-healing assay at 24 h post-transfection; clonogenic survival assays were performed to determine survival fractions after transfection combined with 2 Gy irradiation, with cells seeded at different densities in 6-well plates (500 cells/well for pcDNA3.1+, oeTSPAN32, and oe+siTSPAN32 groups; 1000 cells/well for pcDNA3.1++2 Gy, oeTSPAN32 + 2 Gy, and oe+siTSPAN32 + 2 Gy groups), followed by 12 days of culture, fixation with 4% paraformaldehyde, and staining with 1% crystal violet; acute cell death was quantified by flow cytometry using the Annexin V-FITC/PI apoptosis detection kit (BD Biosciences, 556547) at 48 h after transfection combined with 4 Gy irradiation.

### 2.11 RNA extraction and RT-qPCR assays

A549 and H1299 cells were transfected with TSPAN32 overexpression plasmids or siRNA interference fragments, respectively. Cell samples were collected after 24 h of incubation. Meanwhile, tumor tissues were dissected from nude mice bearing TSPAN32-overexpressing cell-derived tumors following X-ray irradiation treatment. Total RNA was extracted using the RNA-Quick Purification Kit (YISHAN, Shanghai, China) and was reverse-transcribed into cDNA using HiScript II Q RT SuperMix for qPCR (+gDNA wiper) (Vazyme, Jiangsu, China). Real-time quantitative PCR (RT-qPCR) was performed using ChamQ Blue Universal SYBR qPCR Master Mix (Vazyme, Jiangsu, China). *β-actin* was used as the reference gene. The Primer sequences were listed in **Supplementary Table 7**.

### 2.12 Western blotting analysis

A549 and H1299 cells were transfected with TSPAN32 overexpression plasmid alone or combined with X-ray irradiation treatment. Cell samples were collected 48 h later, and total protein was extracted using RIPA lysis buffer (Beyotime, P0038). Protein samples were separated by SDS-PAGE and transferred to PVDF membranes. The membranes were blocked with 5% skim milk at room temperature for 1 h, followed by overnight incubation with primary antibodies at 4 °C. The primary antibodies included: TSPAN32 (1:1000, 20320-1-AP, Proteintech), Phospho-PTEN (Thr366) (1:1000, F2618, Selleck), pan-AKT (1:1000, ab8805, Abcam), Phospho-AKT (Ser473) (1:1000, 66444-1-lg, Proteintech), mTOR (1:1000, 66888-1-Ig, Proteintech), GAPDH (1:1000, 60004-1-Ig, Proteintech). HRP-conjugated anti-rabbit or anti-mouse secondary antibodies (CST) were used as secondary antibodies.

### 2.13 Co-immunoprecipitation

TSPAN32 was overexpressed in HEK-293T cells, and total cellular protein was extracted 24 h post-overexpression. For each sample, 500 μg of protein was incubated with 2 μg of anti-TSPAN32 antibody or isotype-matched IgG at 4 °C under gentle rotation overnight; on the following day, 20 μL of Protein A/G agarose beads (sc-2003, Santa Cruz Biotechnology) was added and incubation continued for 2 h. Proteins from the INPUT, IgG, and bead groups were denatured at 95 °C, resolved by SDS-PAGE, and transferred to PVDF membranes. The antibodies used were TSPAN32 (1:1000, 20320-1-AP, Proteintech) and Phospho-PTEN (Thr366) (1:1000, F2618, Selleck).

### 2.14 Animal experiments

Animals: Female BALB/c nude mice (nu/nu), aged 3–8 weeks, were purchased from GemPharmatech Co., Ltd. (license no. SCXK [Guangdong] 2020-0054). The animals were housed under specific-pathogen-free (SPF) conditions at 20-26 °C and 40%-70% relative humidity, with a 12 h light/dark cycle and were provided ad libitum access to sterilized food and water. The animal study protocol was approved by the Institutional Animal Care and Use Committee of the Cancer Center of Sun Yat-sen University (Protocol No. L025501202507010, July 23, 2025).

A total of 30 female BALB/c nude mice aged 4–8 weeks were used in this study and randomly divided into 4 groups: pcDNA3.1+ group (n=7), oeTSPAN32 group (n=7), pcDNA3.1++6 Gy group (n=8), and oeTSPAN32 + 6 Gy group (n=8). A 20% dropout margin was reserved (covering anesthesia accidents, post-irradiation deaths, and sample collection failures). Ultimately, 4 mice from each group that completed the experiment with complete data were included in the statistical analysis.

The nude mice were acclimatized for one week prior to the experiments. A549 cells and TSPAN32-overexpressing A549 cells in the logarithmic growth phase were resuspended in PBS, and 1×10^7^ cells per mouse were subcutaneously inoculated into the right hind limb of the dorsal back of nude mice. Tumor length (a) and width (b) were measured every two days, and tumor volume was calculated using the formula V = 0.5 × a × b². When the average tumor volume of the control group reached approximately 200 mm³ (approximately 15 days post-inoculation), the nude mice were subjected to X-ray irradiation (6 Gy, single dose). Prior to irradiation, mice were anesthetized with isoflurane and immobilized in a lead shielding device, with only the tumor area exposed while the rest of the body was shielded with lead plates. Tumor volume was measured every two days after irradiation. When the tumor volume of the control group reached 1000 mm³, the nude mice were euthanized by CO_2_ asphyxiation, and tumor tissues were completely dissected, weighed, and photographed.

### 2.15 Statistical analysis

R (version 4.2.1) and the GraphPad Prism 8 software were applied for statistical analysis. A Student’s t-test was employed to analyze the expression and the distribution of risk score, stromal score, and immune score across different groups. For multiple comparisons among three groups, one-way analysis of variance (ANOVA) was used. All experiments were performed at least thrice. Statistical significance was set at P < 0.05.

## 3 Results

### 3.1 Identification of differentially expressed genes associated with radiotherapy efficacy in lung adenocarcinoma

**Figure 1:**
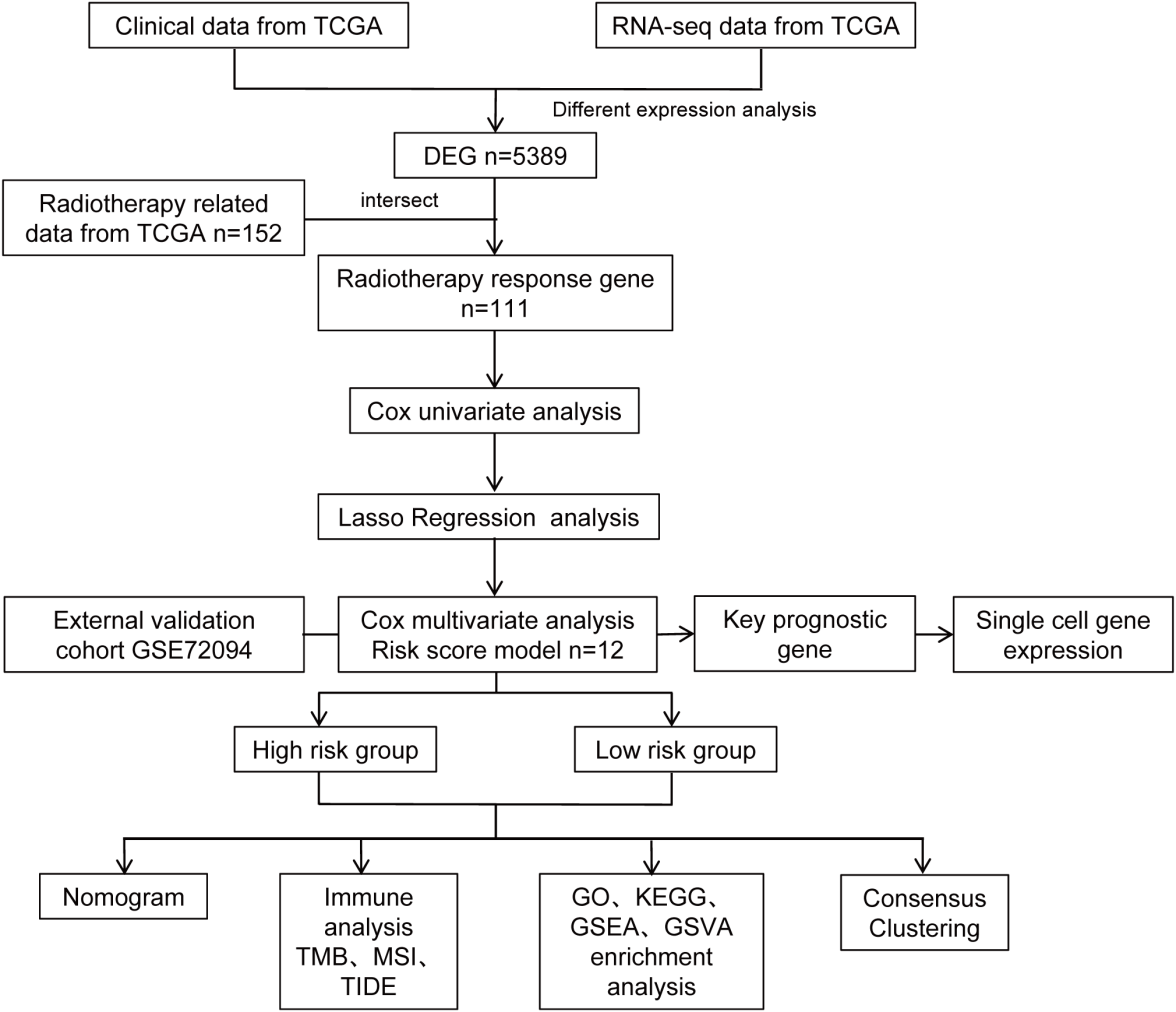
Workflow for model development in this study.

**Figure 2:**
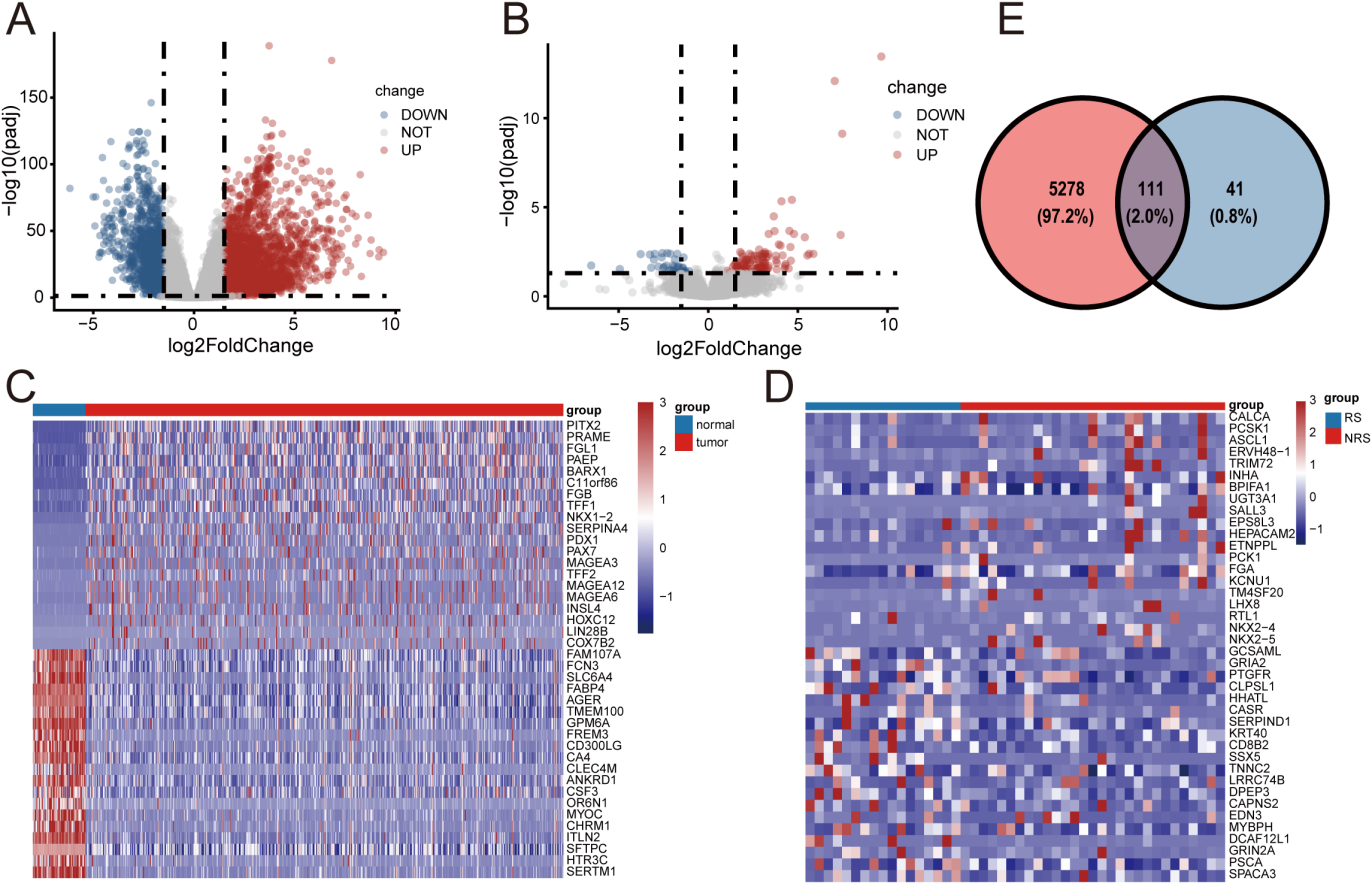
Identifies differentially expressed genes associated with radiotherapy efficacy based on the TCGA-LUAD cohort **(A)**. The volcano plot illustrates the overall distribution of 5,389 differentially expressed genes (DEGs) between tumor tissues and normal tissues in the TCGA-LUAD cohort. **(B)**. The volcano plot shows the distribution of 152 DEGs in tumor samples from the disease remission group (responder) and the disease progression group (non-responder) following radiotherapy. **(C)**. The heatmap compares the expression patterns of the top 20 upregulated genes and the top 20 downregulated genes between tumor tissues and normal tissues. **(D)**. The heatmap compares the expression patterns of the top 20 upregulated genes and the top 20 downregulated genes between the disease remission group and the disease progression group following radiotherapy. **(E)**. The Venn diagram reveals the intersection of 5,389 tumor-normal DEGs and 152 radiotherapy response DEGs, identifying 111 candidate genes associated with radiotherapy efficacy.

The overall workflow of this study is shown in **Figure 1**. Initially, 5,389 differentially expressed genes (DEGs) were identified by comparing the gene expression profiles between the normal and tumor groups (**Figures 2A, C**; **Supplementary Table 1**). Subsequently, 152 DEGs were screened out by comparing the gene expression profiles between the radiotherapy-responsive and non-responsive groups (**Figures 2B, D**; **Supplementary Table 2**). Based on these findings, a joint analysis was conducted on the 5,389 DEGs identified between the normal and tumor groups and the 152 DEGs identified between the radiotherapy-responsive and non-responsive groups. This analysis ultimately led to the identification of 111 candidate DEGs associated with radiotherapy response in lung adenocarcinoma (LUAD) for further analysis (**Figure 2E**).

### 3.2 Model construction

**Figure 3:**
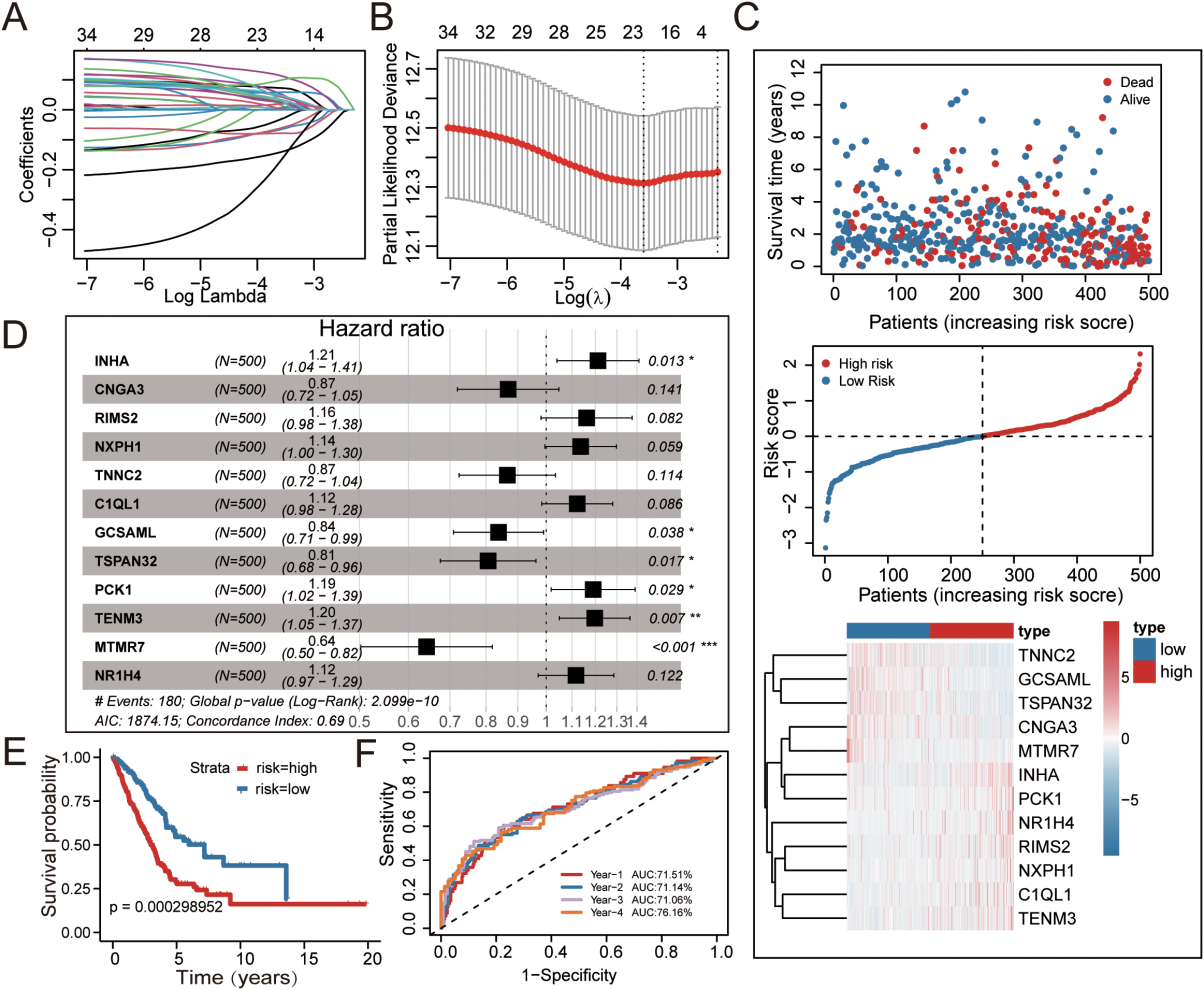
Evaluation of the prognostic risk model in the training cohort. **(A, B)** LASSO regression identified 35 overall survival-associated genes. The tuning parameter (λ) was optimized via cross-validation. The x-axis shows log(λ); the y-axis shows the deviance. The red dots indicate the mean deviance (± standard error). **(C)** Distribution of patient data in the TCGA-LUAD training cohort. From top to bottom: (1) Patient survival status (red dots: deceased; blue dots: alive); (2) Calculated risk scores; (3) Expression heatmap of the 12 prognostic genes. **(D)** Multivariate Cox regression identified 12 genes significantly associated with lung adenocarcinoma (LUAD) patient prognosis. **(E)** Kaplan-Meier curves for OS of high-risk and low-risk groups in the TCGA-LUAD training cohort. **(F)** Receiver operating characteristic (ROC) curves for predicting 1-, 2-, 3-, and 4-year OS in the TCGA-LUAD training cohort.

Based on the 111 radiotherapy response-associated DEGs, univariate COX regression identified 35 genes as potential prognostic biomarkers in LUAD (p < 0.05, **Supplementary Table 3**). Subsequently, these candidate genes were further refined to 24 via LASSO regression and then to 12 by multivariate COX regression (**Figures 3A–D**; **Supplementary Table 4**, **Supplementary Table 5**). Ultimately, a prognostic model for LUAD patients was constructed based on these 12 genes (*INHA, CNGA3, RIMS2, NXPH1, TNNC2, C1QL1, GCSAML, TSPAN32, PCK1, TENM3, MTMR7* and *NR1H4*) (**Table 1**).

**Table 1:** A list of the 12 key differentially expressed genes from the model.

Gene symbol	Gene ID	Full name	Function of the encoded protein (Originating from the UniProt and NCBI databases)
*INHA*	3623	INHA inhibin subunit alpha	This gene encodes a member of the TGF-beta (transforming growth factor-beta) superfamily. Its product, inhibin, negatively regulates the secretion of follicle-stimulating hormone from the pituitary gland and is involved in regulating various cellular processes, including cell proliferation, apoptosis, immune response, and hormone secretion.
*CNGA3*	1261	cyclic nucleotide gated channel subunit alpha 3	This gene encodes a member of the cyclic nucleotide-gated cation channel protein family which is required for normal vision and olfactory signal transduction.
*RIMS2*	9699	regulating synaptic membrane exocytosis 2	This gene encodes a presynaptic protein that interacts with RAB3 and other synaptic proteins (e.g., UNC-13B, ELKS, synaptotagmin 1), playing a role in synaptic exocytosis. Its polymorphisms are linked to degenerative lumbar scoliosis.
*NXPH1*	30010	neurexophilin 1	This gene encodes a neurexophilin family secreted protein with variable N-terminal, conserved N-glycosylated central, and cysteine-rich C-terminal domains. It forms a tight complex with alpha neurexins, which promote dendrite-axon adhesion.
*TNNC2*	7125	troponin C2, fast skeletal type	Troponin (Tn), a key regulator of striated muscle contraction, consists of three subunits: Tn-I (inhibits actomyosin ATPase), Tn-T (binds tropomyosin and Tn-C), and Tn-C (binds calcium and relieves inhibition on actin filaments). This gene encodes the Tn-C subunit.
*C1QL1*	10882	complement C1q like 1	Predicted to enable signaling receptor binding, regulate locomotory behavior, and act in synapse maintenance, motor learning, and neuron remodeling. Located in climbing fiber, extracellular region, and presynapse; part of collagen trimer; active in cerebellar climbing fiber-Purkinje cell synapse and synaptic cleft.
*GCSAML*	148823	germinal center associated signaling and motility like	This gene encodes a protein thought to be a signaling molecule associated with germinal centers, the sites of proliferation and differentiation of mature B lymphocytes. Alternate splicing results in multiple transcript variants.
*TSPAN32*	10077	tetraspanin 32	This gene, a tetraspanin family member, is a tumor-suppressing fragment in the chromosome 11p15.5 imprinted domain, linked to Beckwith-Wiedemann syndrome, Wilms tumor, rhabdomyosarcoma, adrenocortical carcinoma, and lung, ovarian, and breast cancers. It escapes imprinting and may function in malignancies, diseases, and hematopoiesis. Alternative splicing variants exist, but their biological roles are unclear.
*PCK1*	5105	phosphoenolpyruvate carboxykinase 1	This gene regulates gluconeogenesis, encoding a cytosolic enzyme that converts oxaloacetate to phosphoenolpyruvate using GTP, releasing CO_2_ and GDP. Its expression is controlled by insulin, glucocorticoids, glucagon, cAMP, and diet. Defects lead to cytosolic PEPCK deficiency. A mitochondrial isozyme has also been identified.
*TENM3*	55714	teneurin transmembrane protein 3	This gene encodes a teneurin transmembrane protein family member, potentially regulating neuronal development, including the visual pathway. Mutations are linked to microphthalmia and hip dysplasia.
*MTMR7*	9108	myotubularin related protein 7	This gene encodes a myotubularin family tyrosine/dual-specificity phosphatase with four conserved domains: glucosyltransferase, Rab-like GTPase activator/myotubularins, Rac-induced recruitment, and protein tyrosine/dual-specificity phosphatases, along with a SET interaction domain. It dephosphorylates phosphatidylinositol 3-phosphate and inositol 1,3-bisphosphate.
*NR1H4*	9971	nuclear receptor subfamily 1 group H member 4	This gene encodes a ligand-activated transcription factor resembling nuclear hormone receptors. It acts as a bile acid receptor, binding to DNA to regulate bile acid synthesis and transport genes when activated. Various alternatively spliced isoforms exist.

In the training and validation cohorts, the risk score for each lung cancer patient was calculated using the following formula:


$$\begin{array}{l} Risk\;score\;=\;0.192*INHA－0.141*CNGA3+0.150*RIMS2\\ +0.128*NXPH1－0.144*TNNC2\\ +0.115*C1QL1－0.177*GCSAML－0.215*TSPAN32\\ +0.174*PCK1+0.180*TENM3－0.444*MTMR7\\ +0.111*NR1H4 \end{array}$$

Patients were stratified into high-risk and low-risk subgroups based on the median risk score. Kaplan-Meier survival curves demonstrated that the high-risk subgroup had significantly worse overall survival (OS) than the low-risk subgroup (p = 0.0002, **Figure 3E**). The prognostic model demonstrated robust predictive accuracy for 1-, 2-, 3-, and 4-year OS, with area under the curve (AUC) values of 71.51%, 71.14%, 71.06%, and 71.16%, respectively (**Figure 3F**). Additionally, the relationships between risk scores, survival time, survival status, and risk levels, as well as the heatmap of the expression levels of the 12 genes, are shown in **Figure 3C**. Collectively, these results indicate that the risk model constructed in this study exhibits robustness in predicting the prognosis of LUAD patients.

**Figure 4:**
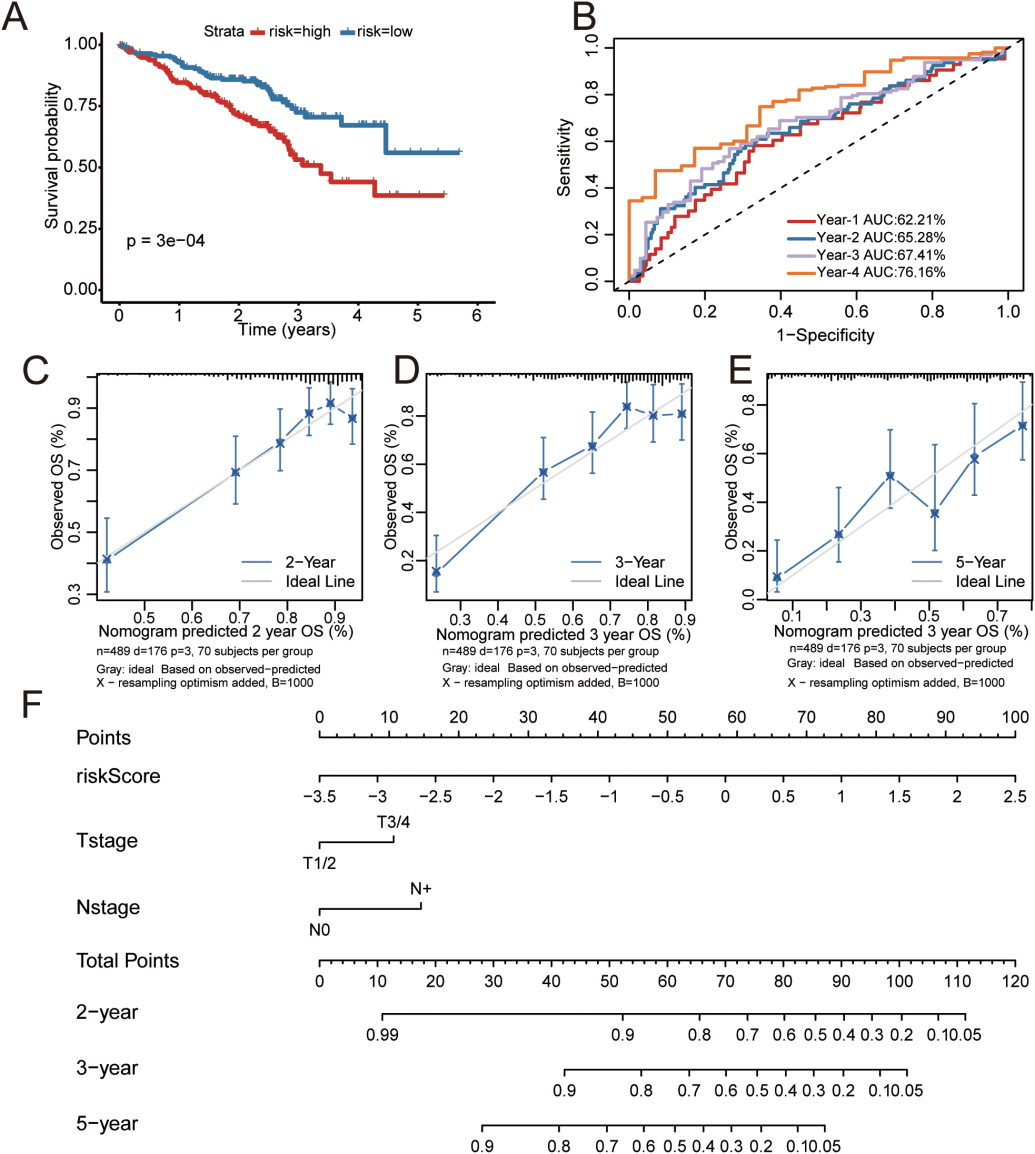
Prognostic model and nomogram validation in lung adenocarcinoma. **(A)** Kaplan-Meier curves for OS of high-risk and low-risk groups in the GSE72094 validation dataset. **(B)** Receiver operating characteristic curves for predicting 1-, 2-, 3-, and 4-year OS in the GSE72094 validation dataset. **(C-E)** Calibration curves of the nomogram for 2-, 3-, and 5-year overall survival (OS) in the training cohort of lung adenocarcinoma patients. The gray line indicates the ideal reference line where predicted probabilities match the observed survival rates. **(F)** Nomogram for the main prognostic indicators in the training cohort.

The prognostic model was robustly validated in the independent GSE72094 cohort. High-risk patients had significantly worse overall survival, consistent with the training cohort (**Figure 4A**). ROC analysis yielded area under the curve (AUC) values of 62.21%, 65.28%, 67.41%, and 76.16% for 1-, 2-, 3-, and 4-year survival, respectively (**Figure 4B**). A nomogram that integrated the risk score with all significant clinical features was developed for quantitative prognosis prediction and clinical decision-making (28). Analysis of the nomogram demonstrated that the risk score was the dominant determinant of prognosis (**Figure 4F**). Calibration curves indicated close agreement between observed and nomogram-predicted survival probabilities at 2-, 3-, and 5-year (**Figures 4C–E**). These results collectively validate the robustness and clinical utility of the model.

### 3.3 Risk score correlates with immune signatures and therapy response in LUAD

**Figure 5:**
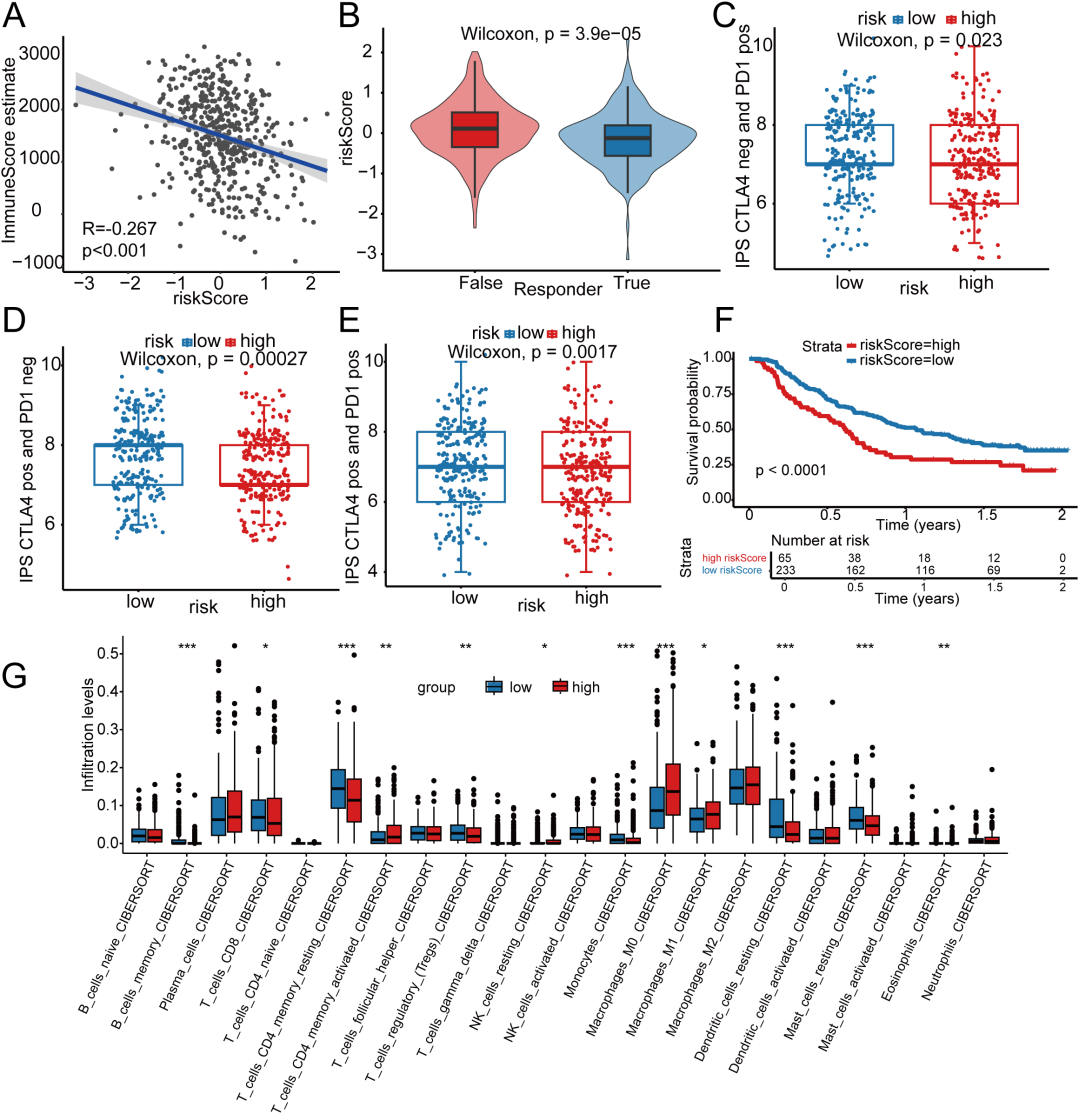
Immune-characteristic comparison between TCGA-LUAD low- and high-risk groups (divided by median risk score). **(A)** Correlation analysis between risk score and Stromal Score. **(B)** Evaluation of tumor immune dysfunction and exclusion using TIDE (Tumor Immune Dysfunction and Exclusion) score. **(C-E)** Analysis of immunogenicity scores based on TCIA (The Cancer Immunome Atlas). **(F)** Kaplan-Meier curve analysis of OS in patients with high and low risk after immunotherapy. **(G)** CIBERSORT reveals distinct immune-cell infiltration patterns between low- and high-risk TCGA-LUAD groups. * p < 0.05, ** p < 0.01, *** p < 0.001.

The tumor immune microenvironment shapes therapy response and prognosis. We first examined how the risk score correlates with the TME. As shown in **Figure 5A**, the risk score in LUAD was negatively correlated with the stromal score (correlation coefficient = -0.267, p = 1.34×10^-9^). The stromal score was significantly higher in the low-risk group, indicating a potentially greater sensitivity to immunotherapy. TIDE was then used to quantify T-cell dysfunction and exclusion, predicting response to immune checkpoint inhibitors. The analysis indicated a significantly higher TIDE score in non-responders (responder false) than in responders (responder true) (p < 0.001) (**Supplementary Table 6**). This points to a higher likelihood of immune escape in radiotherapy-insensitive tumors, correlating with a reduced benefit from immunotherapy in these patients. Comprehensive analysis suggested that the low-risk group was more responsive to immunotherapy, whereas the high-risk group showed minimal benefit (**Figure 5B**). To validate this, we utilized The Cancer Immunome Atlas (TCIA) to calculate the Immunophenoscore (IPS), which predicts response to cytotoxic T-lymphocyte-associated antigen-4 (CTLA-4) and programmed cell death protein 1 (PD-1) blockade. Indeed, the low-risk group exhibited significantly higher IPS for both CTLA-4 and PD-1 positivity (**Figures 5C–E**), further supporting its greater potential benefit from such therapies. Survival analysis further confirmed that the low-risk group had a superior prognosis following immunotherapy (**Figure 5F**). Consistent with the TIDE and TCIA findings, these results collectively suggest that patients who are responsive to radiotherapy (typically the low-risk group) may derive enhanced benefit from combined immunotherapy.

The CIBERSORT algorithm (100 iterations) was used to compare immune-cell infiltration between high- and low-risk groups. The results showed that the levels of “B cells memory”, “T cells CD8”, “T cells CD4 memory resting”, “T cells regulatory (Tregs)”, “Monocytes”, “Dendritic cells resting”, and “Mast cells resting” were significantly higher in the low-risk group (**Figure 5G**). This profile suggests a state of potent yet regulated anti-tumor immunity: strong memory and cytotoxic potential enable effective tumor antigen recognition and killing, while regulatory components and resting antigen-presenting cells may maintain homeostasis and prevent excessive inflammation, collectively contributing to a more favorable prognosis. In contrast, the levels of “Eosinophils”, “T cells CD4 memory activated”, “NK cells resting”, and “Macrophages M0” are significantly elevated in the high-risk group (**Figure 5G**). This profile depicts a dysregulated and pro-tumorigenic immune microenvironment: it is marked by overt inflammation, impaired macrophage differentiation (Macrophages M0), functional inactivation of innate effectors (NK cells resting), and potentially dysfunctional T-cell activation. Rather than mounting an effective anti-tumor response, this environment may sustain chronic inflammation that causes tissue damage and immune suppression while failing to eliminate tumor cells. Consequently, such a functionally disrupted TME likely facilitates tumor immune evasion, invasion, and metastasis, ultimately driving disease progression and poor prognosis.

### 3.4 *TSPAN32* is a potential prognostic marker for radiotherapy combined with immunotherapy in LUAD

**Figure 6:**
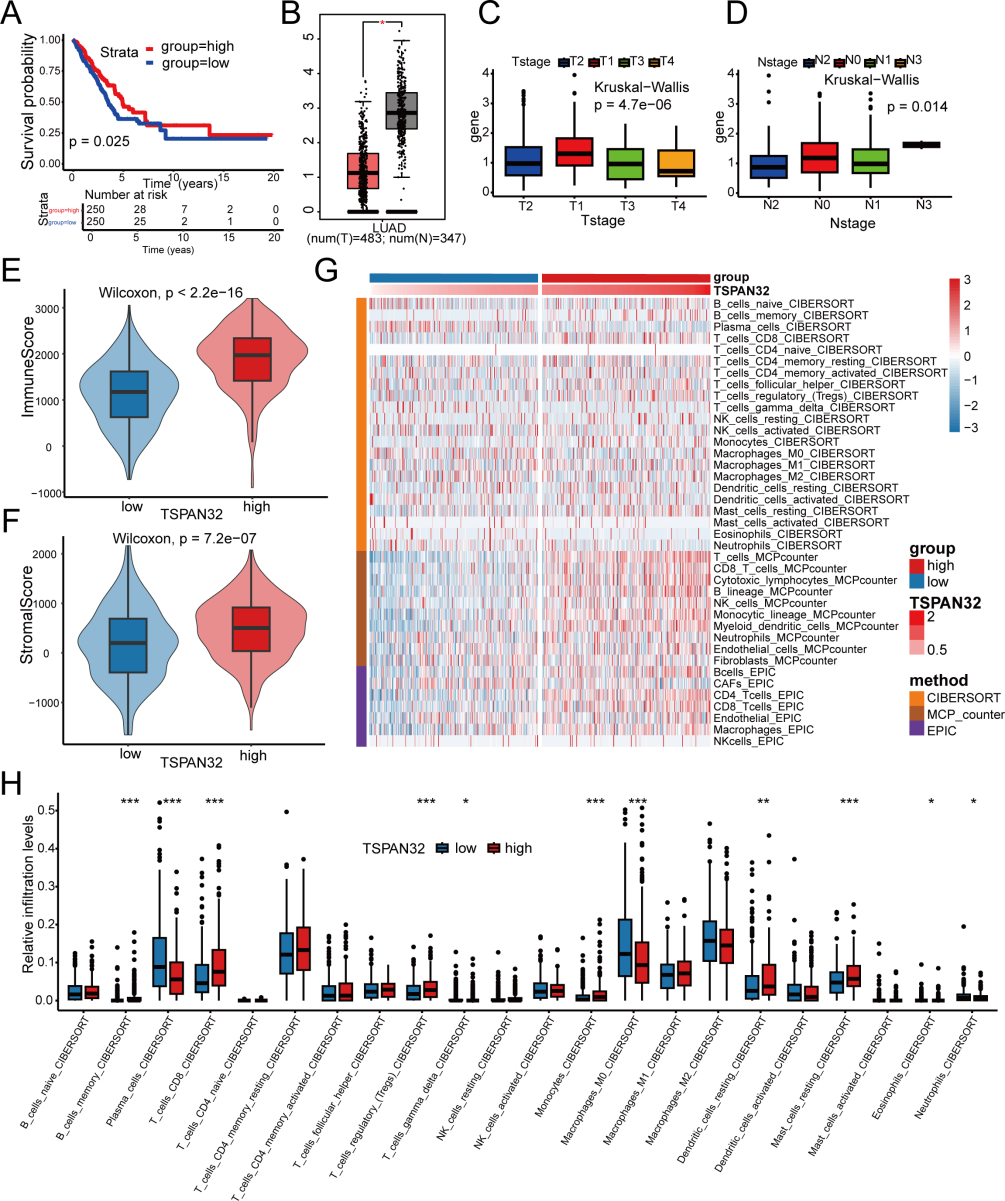
Association of *TSPAN32* with clinical features, survival, and immune infiltration. **(A)** Kaplan-Meier curve for predicting the OS rate between the *TSPAN32* low- and high-expression group. **(B)** Expression level of *TSPAN32* in LUAD. **(C)** Correlation between *TSPAN32* expression level and clinical T stage. **(D)** Correlation between *TSPAN32* expression level and clinical N stage. **(E)** Distribution of immune scores between the *TSPAN32* low- and high-expression group. **(F)** Distribution of stromal scores between the *TSPAN32* low- and high-expression group. **(G)** Heatmap of immune-related pathway activity correlates with *TSPAN32* expression groups (red: high; blue: low). **(H)** CIBERSORT analysis of immune-cell abundance differences between low- and high-TSPAN32 groups. * p < 0.05, ** p < 0.01, *** p < 0.001

The 12-gene risk score model assigns a relatively high coefficient to *TSPAN32*, indicating its key contribution. Given this weight and its close association with the immune system, *TSPAN32* emerges as a promising biomarker. While multi-gene models enhance robustness, analyzing *TSPAN32* expression separately can help clarify the molecular mechanisms linking radiotherapy sensitivity to immunotherapy response. Analysis of 31 TCGA cancer types via the GEPIA2 database (Gene Expression Profiling Interactive Analysis) showed that *TSPAN32* is significantly downregulated in multiple malignancies, including LUAD and lung squamous cell carcinoma (http://gepia2.cancer-pku.cn/). Moreover, its low expression correlates significantly with poor patient prognosis (**Figure 6A**). Nevertheless, *TSPAN32* expression was heterogeneous across driver-gene subtypes. TSPAN32 was significantly up-regulated in both EGFR-mutant and KRAS-mutant LUAD versus wild-type (both p < 0.05) (**Supplementary Figures 2A, D**). Among EGFR-mutant patients, high *TSPAN32* predicted longer overall survival (p < 0.05) (**Supplementary Figures 2B, C**), whereas no association was seen in the KRAS-mutant group (p > 0.05) (**Supplementary Figures 2E, F**), indicating that the prognostic value of *TSPAN32* is modulated by driver-gene context. In LUAD, *TSPAN32* expression correlates significantly with T stage (**Figure 6C**) and N stage (**Figure 6D**), linking it to clinicopathological severity and treatment response. These findings validate our hypothesis and offer a basis for investigating *TSPAN32*’s specific role in LUAD pathogenesis and treatment response. However, the model’s limited predictive power in KRAS-mutant cases indicates a need for further optimization to enhance stratification accuracy.

Integrated analysis (CIBERSORT, ESTIMATE, and MCP-counter) revealed that the TSPAN32-high group exhibits an “immune-hot” tumor microenvironment, characterized by significantly elevated infiltration of Memory B cells, CD8^+^ T cells, NK cells, and Monocytes (**Figure 6G**). This profile was marked by high immune and stromal scores (both p < 0.001; **Figures 6E, H**) and a prominent CD8^+^ T cell presence (**Figure 6H**). Further characterization using CIBERSORT confirmed the enrichment of specific immune-activating subsets, including B cells memory, T cells CD8, Regulatory T cells, Monocytes, Activated Dendritic cells, and Resting mast cells (all p < 0.05). Besides *TSPAN32*, the other 11 genes in the model also showed significant correlations with stromal and immune scores. For instance, high expression of *GCSAML* and *TENM3*, as well as low expression of *MTMER7*, *INHA*, and *NXPH1*, were all associated with elevated scores. This collective synergy among the genes forms the biological foundation of the robust risk model (**Supplementary Figure 3**, **Supplementary Figure 4**). Systematic GO and KEGG enrichment analyses of these 11 genes mapped them to an integrated network of “cell metabolism-immune microenvironment interaction and regulation”. They were significantly enriched in pathways related to metabolic reprogramming (KEGG) and biological processes such as “negative regulation of interferon-gamma production” and “hormone secretion” and “signal release” (GO). Thus, the model’s robustness rests on a multi-gene synergy in which *TSPAN32* is central, promoting immune-cell recruitment and activation. Its expression positively associates with clinical response, offering a promising biomarker for immunotherapy efficacy and personalized treatment decisions.

### 3.5 *TSPAN32* overexpression curbs LUAD proliferation and migration while enhancing radiosensitivity

The expression of *TSPAN32* was found to be significantly downregulated in LUAD cell lines compared to non-tumor samples, as confirmed by Real-time quantitative PCR (RT-qPCR) and Western blotting (WB) analyses (**Supplementary Figure 6A**). Based on its low endogenous levels, A549 and H1299 cells were selected for functional studies (**Supplementary Figure 6B**).

**Figure 7:**
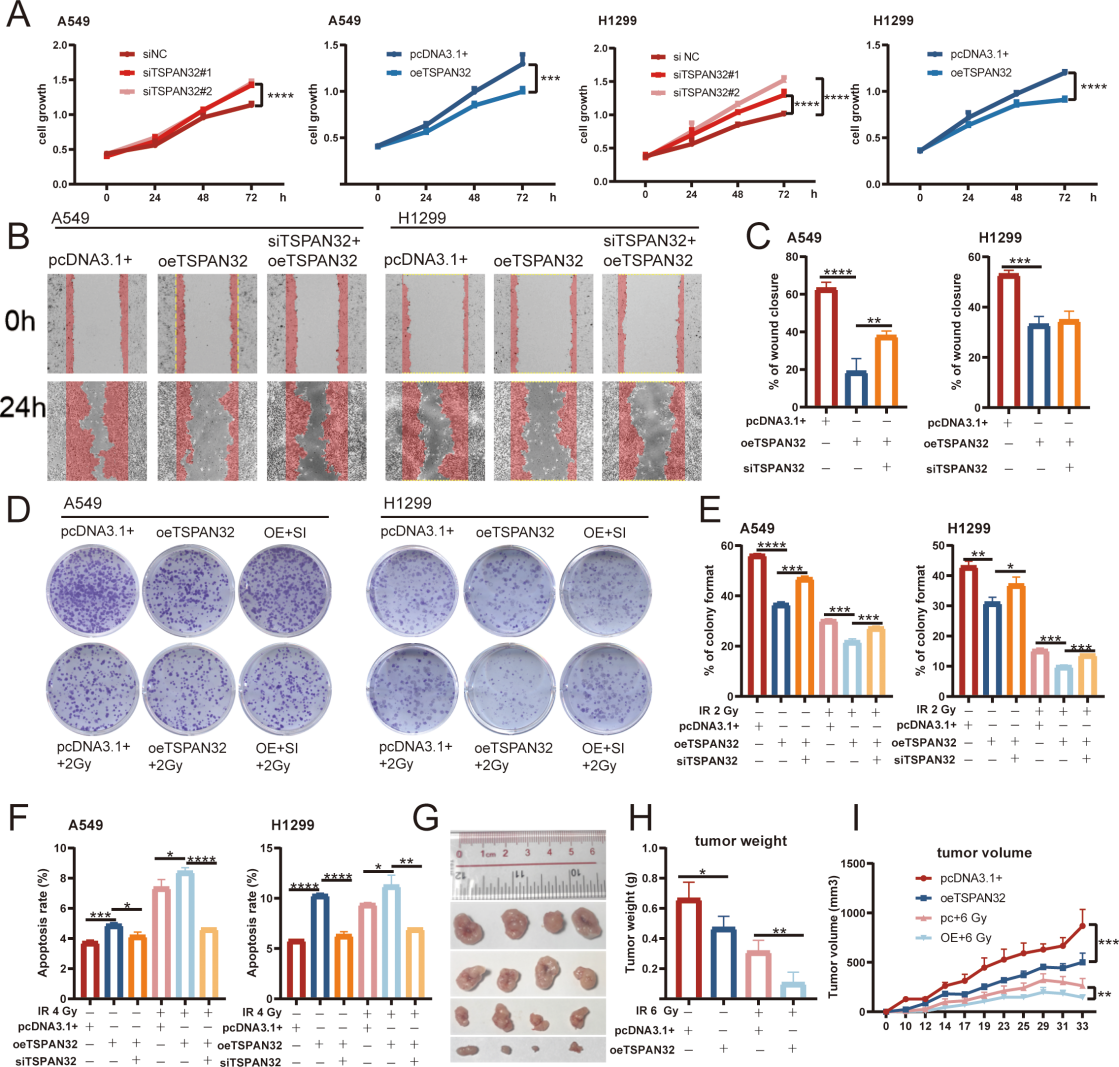
Overexpression of *TSPAN32* inhibits proliferation and migration of lung cancer cells and enhances their radiosensitivity. **(A)** 3-(4,5-Dimethylthiazol-2-yl)-2,5-diphenyltetrazolium bromide (MTT) assay showing *TSPAN32* knockdown (si-TSPAN32) promotes while overexpression (oe-TSPAN32) suppresses A549 and H1299 cell proliferation. **(B, C)** Wound healing assay revealing the effect of TSPAN32 on cell migration. B: Representative images of wound closure in A549 and H1299 cells at 24 h after wounding; C: Quantification. **(D, E)** Clonogenic assay: surviving fraction after irradiation in TSPAN32-overexpressing vs control cells. D: Representative images; E: Quantification. **(F)** Apoptosis in A549 and H1299 cells after *TSPAN32* modulation and 4 Gy X−ray irradiation. **(G-I)** Subcutaneous A549 xenografts: *TSPAN32* overexpression markedly slowed tumor growth. G: Representative tumors; H: Final tumor weights; I: Growth curves over time. pcDNA3.1+ serves as the control group; oe-TSPAN32 refers to the overexpression group, abbreviated as OE; si-TSPAN32 represents the TSPAN32-targeted interference group (small interfering RNA), abbreviated as SI. * p < 0.05, ** p < 0.01, *** p < 0.001.

To investigate the effect of TSPAN32 on tumor radiosensitivity in LUAD, the roles of TSPAN32 in regulating the growth, proliferation, and invasion of LUAD cells were examined. TSPAN32 was knocked down or overexpressed in A549 and H1299 cells, respectively. MTT cell proliferation assays showed that overexpression of the TSPAN32 gene (oe-TSPAN32) significantly inhibited cell proliferation (**Figure 7A**), whereas knockdown of TSPAN32 (si-TSPAN32) promoted cell proliferation (**Figure 7A**). The study further investigated whether changes in *TSPAN32* expression affect cell migration capacity. Wound healing assay results demonstrated that overexpression of *TSPAN32* significantly impaired the wound closure ability of these cells, while knockdown of *TSPAN32* effectively restored their migratory capacity (**Figures 7B, C**). To further validate the tumor-suppressive potential of TSPAN32 *in vivo*, TSPAN32-overexpressing A549 cells were subcutaneously injected into female BALB/c nude mice. The results revealed that TSPAN32-overexpressing tumors exhibited significantly slower growth rates compared with the negative control group (**Figures 7G, H**). These findings collectively demonstrate that TSPAN32 functions as a tumor suppressor in LUAD by curbing tumor cell proliferation and migration.

The regulatory role of TSPAN32 in the radiosensitivity of lung cancer cells was systematically evaluated through radiation sensitivity experiments. The experiments employed wild-type and genetically modified A549 and H1299 cell lines, including the si-TSPAN32 knockdown group and the oe-TSPAN32 overexpression group, which were irradiated with a single dose of 2, 4, or 6 Gy X-ray. The results showed that: (1) Compared with the control group, cells in the A549 oe-TSPAN32 group exhibited a significantly increased acute mortality rate after 4 Gy X-ray irradiation (p < 0.05), while this effect was significantly attenuated after si-TSPAN32 treatment (p < 0.01) (**Figure 7F**). (2) Clone formation experiments further confirmed that the long-term survival rate of cells in the A549 oe-TSPAN32 group was significantly reduced after 2 Gy X-ray irradiation (the clone formation rate decreased by 34.3 ± 1.2% compared with the control group, p < 0.001), and this radiosensitizing effect could be reversed by TSPAN32 knockdown (the clone formation rate decreased by 16.0 ± 1.7% compared with the control group, p < 0.05) (**Figures 7D, E**). A similar trend was observed in the H1299 cell line. *In vivo* experiments demonstrated that overexpression of TSPAN32 enhanced the radiosensitivity of A549 cells in tumor formation in nude mice and inhibited tumor growth. As shown in **Figures 7G–I**, compared with the control group, tumors formed by TSPAN32-overexpressing A549 cells exhibited significantly reduced volume growth rates after irradiation. These results consistently indicate that the expression level of TSPAN32 is positively correlated with the radiosensitivity of lung cancer cells, and its overexpression can significantly enhance the sensitivity of tumor cells to radiotherapy, providing experimental evidence for TSPAN32 as a potential target for radiosensitization.

### 3.6 The TSPAN32-PTEN signaling axis affects the cellular immune microenvironment

**Figure 8:**
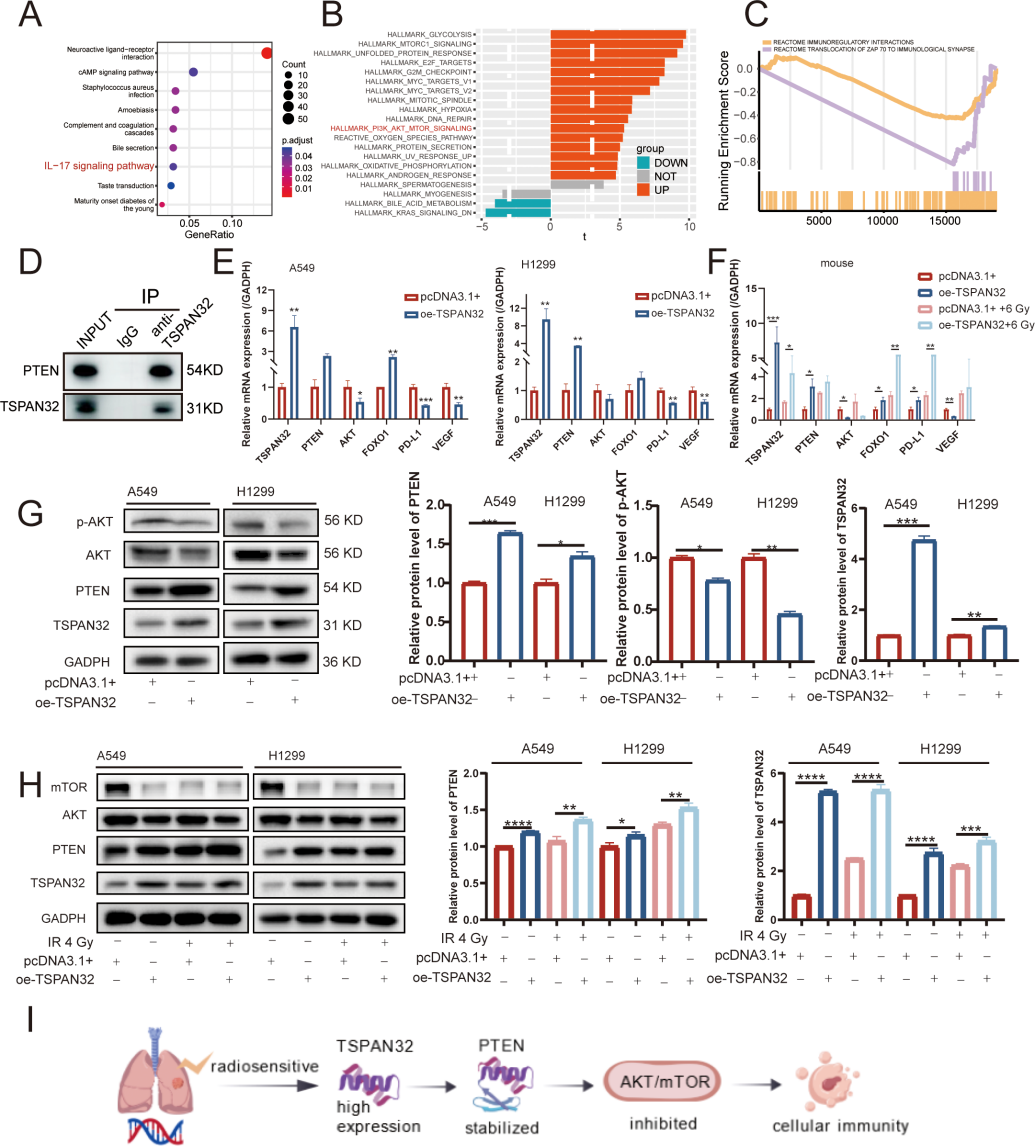
The regulatory mechanism of TSPAN32 in LUAD through stabilization of PTEN. **(A)** KEGG enrichment analysis of differentially expressed genes. **(B)** Gene set enrichment analysis (GSVA) of high- and low-risk groups based on risk scores. Green: low-risk group; Orange: high-risk group. **(C)** GSEA analysis showing pathways enriched in the low-risk group, with significance threshold set at FDR q < 0.05. **(D)** Co-immunoprecipitation data for the TSPAN32-PTEN interaction. **(E)** Real-time quantitative PCR (RT-qPCR) analysis of the expression levels of *TSPAN32, PTEN, AKT, FOXO1, PD-L1*, and *VEGF* genes in A549 and H1299 cells after TSPAN32 overexpression. **(F)** RT-qPCR analysis of the expression levels of *TSPAN32, PTEN, AKT, FOXO1, PD-L1*, and *VEGF* genes in subcutaneous tumor tissues of nude mice. **(G)** Western blotting (WB) analysis of PTEN and AKT protein expression levels in A549 and H1299 cells after TSPAN32 overexpression. **(H)** WB analysis of PTEN, AKT, and mTOR protein expression levels in A549 and H1299 cells after ionizing radiation (4 Gy). **(I)** Schematic diagram illustrating the role of the TSPAN32-PTEN signaling pathway in regulating the sensitivity of LUAD.

KEGG enrichment of the 12 DEGs highlighted the IL-17 signaling pathway (**Figure 8A**). GSVA linked the high-risk signature to AKT-mTOR signaling (**Figure 8B**), whereas GSEA associated the low-risk signature with immune-regulatory pathways (**Figure 8C**). TSPAN32 reportedly binds PTEN, blocking its ubiquitination and stabilizing the protein (18); PTEN in turn dephosphorylates PIP3, antagonizing AKT activation (29) and modulating IL-17 expression (30). We first confirmed the TSPAN32-PTEN interaction by co-immunoprecipitation (**Figure 8D**). RT-qPCR in A549 and H1299 cells showed that TSPAN32 overexpression up-regulated PTEN transcripts (significant in H1299) and down-regulated AKT transcripts (significant in A549) (**Figure 8E**), consistent with axis activation.

Previous studies indicate that AKT promotes FOXO1 nuclear export and proteasomal degradation via phosphorylation, thereby suppressing FOXO1 protein levels (31); FOXO1 in turn regulates IL-17 expression (32). To connect this to our observed IL-17 pathway enrichment, we examined FOXO1. RT-qPCR revealed that TSPAN32 overexpression elevated FOXO1 mRNA, reaching significance in A549 but not in H1299 cells. Additionally, we used WB to detect the changes in PTEN protein expression after TSPAN32 overexpression (**Figure 8G**). The results indicated that TSPAN32 overexpression significantly enhanced the expression of PTEN and significantly inhibited the expression of phosphorylated AKT. Under the condition of TSPAN32 overexpression combined with X-ray irradiation (**Figure 8H**), the expression level of PTEN was upregulated, while the expression levels of AKT and mTOR were downregulated. This suggests that TSPAN32 overexpression may enhance cellular sensitivity to X-ray irradiation by stabilizing PTEN. In summary, this pathway may primarily exert its effects through post-translational modifications rather than at the transcriptional level. These findings suggest that TSPAN32 may participate in the regulation of IL-17 signaling through the PTEN-AKT-FOXO1 axis; however, the direct effect of TSPAN32 on IL-17 expression remains to be validated through further experiments.

We further assessed the impact of TSPAN32 on immune checkpoint and angiogenesis markers. TSPAN32 overexpression significantly downregulated both PD-L1 and VEGF mRNA levels *in vitro* (**Figure 8E**). This downregulation was recapitulated in tumor xenografts, where gene expression trends in TSPAN32-overexpressing tumors mirrored those observed in cultured cells (**Figure 8F**). These collective findings suggest that TSPAN32 remodels the tumor immune microenvironment, likely through its role in stabilizing PTEN and suppressing the downstream AKT pathway (**Figure 8I**). The precise mechanisms underlying this immunomodulatory effect warrant further investigation.

## 4 Discussion

Lung cancer is the malignant tumor with the highest mortality rate. Due to its non-specific early symptoms, the majority of patients are diagnosed at an advanced stage of the disease. Even with treatment, the five-year survival rate remains low. As the predominant subtype of lung cancer, adenocarcinoma of the lung holds significant importance for in-depth research. Although there are various treatment modalities for lung cancer, for patients with lung cancer who are not eligible for surgery, radiotherapy is the main alternative. Statistics show that approximately 30%-50% of lung cancer patients have undergone radiotherapy. Radiotherapy can be used at all stages of disease progression, either as a radical treatment or as an adjuvant therapy. However, radiotherapy also has certain drawbacks. While radiation can damage cancerous tissues, it can also harm normal tissues, leading to non-negligible side effects, such as radiation-induced pneumonitis. Currently, research is focusing on combining radiotherapy with immunotherapy. Studies have demonstrated that the combination of radiotherapy and immunotherapy is highly effective. However, this treatment modality is still in its infancy, and there is an urgent need for research to identify biomarkers that can predict which patients will benefit from the combined radiotherapy and immunotherapy.

In this study, by integrating transcriptomic data with clinical prognostic information, we constructed a risk-scoring model based on 12 differentially expressed genes associated with radiotherapy response. This model not only exhibits good prognostic accuracy (AUC > 0.7) but also shows a significant correlation with the tumor immune microenvironment characteristics, suggesting its potential application value in personalized radiotherapy-immunotherapy combinations. From the perspective of the immune microenvironment, we found that patients in the low-risk group (with overexpression of TSPAN32) exhibited an “immune-activated but controlled” microenvironmental profile, characterized by the coordinated infiltration of CD8^+^ T cells, regulatory T cells, and resting dendritic cells. This immune configuration may help maintain the sustainability of antitumor immune responses while avoiding tissue damage caused by excessive inflammation. In contrast, patients in the high-risk group (with low expression of TSPAN32) displayed an “immune-dysregulated” state, dominated by M0-type macrophages, eosinophils, and functionally suppressed NK cells, indicating potential immune evasion and radioresistance. The TIDE and TCIA algorithms further confirmed that patients in the low-risk group had a better response to immune checkpoint inhibitors and a significantly higher immune prognostic score (IPS), supporting the association between the risk score and immune infiltration. Gene-set enrichment analysis revealed that the differentially expressed genes in this model are related to the IL-17 signaling pathway, immune-related pathways, and the AKT-mTOR signaling pathway, suggesting that these molecular mechanisms may influence the cellular immune microenvironment.

TSPAN32, as a high-weight gene in our model, is closely associated with radiotherapy sensitivity and immunotherapy response in LUAD, underscoring its pivotal role in tumor-immune interactions. TSPAN32 is a member of the tetraspanin family, a group of proteins that form membrane microdomains to regulate cell migration, adhesion, signal transduction, and immune response (20, 33), and it shares structural and functional homology with other members. For instance, Tspan15 enhances tumor invasion via ERK phosphorylation (34), and Tspan31 promotes proliferation through the AKT pathway (35). This family is deeply implicated in immunomodulation, influencing antigen presentation, T-cell function, and cytokine production (20, 23, 25, 33), with some members like TSPAN24 serving as therapeutic targets (36, 37) or diagnostic biomarkers (38–40). Our study extends this paradigm to LUAD. We found TSPAN32 significantly downregulated, with low expression correlating with advanced stage and poor prognosis, suggesting a tumor-suppressive role. Functionally, TSPAN32 overexpression inhibited migration, growth, clonogenic survival, and enhanced radiosensitivity in A549 and H1299 cells. Mechanistically, we demonstrated that TSPAN32 stabilizes PTEN, thereby suppressing the AKT/mTOR pathway (**Figure 7H**) and downregulating immune suppressive molecules PD-L1 and VEGF. This aligns with and extends previous findings on the TSPAN family’s role in immune regulation. The immunomodulatory impact of TSPAN32 is further evidenced by our bioinformatic and experimental data: its expression correlates with immune/stromal scores and the abundance of T cells, B cells, and immune checkpoints. Notably, TSPAN32 overexpression promoted infiltration of CD8^+^ T cells, Memory B cells, and Dendritic cells. This result indicates that TSPAN32 may synergize with radiation-induced immunogenic cell death by enhancing antigen presentation and effector T cell activity, thereby influencing the tumor microenvironment through systemic immune modulation and improving patient prognosis. Given its functional overlap with biomarker family members (38–40), TSPAN32 itself emerges as a promising candidate biomarker for stratifying LUAD patients who may benefit from combined radiotherapy and immunotherapy.

Beyond TSPAN32, the remaining 11 genes in the model exhibited significant associations between their expression differences (high vs. low) and stromal/immune scores (**Supplementary Figure 3**, **Supplementary Figure 4**), supporting the model’s relevance to the radiotherapy−immunotherapy interface. Systematic GO and KEGG enrichment analyses of these 11 genes (**Supplementary Figures 5A, B**) revealed their collective mapping to an integrated “cell metabolism−immune microenvironment interaction” network. KEGG analysis highlighted significant enrichment in metabolic reprogramming pathways. This suggests that active glycolysis and mitochondrial metabolism not only fuel tumor proliferation but also directly enhance radiosensitivity by altering redox balance and producing metabolites (e.g., lactate, lipid mediators) that modulate oxidative stress and DNA damage responses. Correspondingly, GO analysis showed enrichment in processes such as “negative regulation of interferon−gamma production”, “hormone secretion”, and “signal release”, thereby linking metabolism to immune regulation. Tumor−derived metabolic products can, for instance via the PPAR pathway, act as signaling molecules to directly regulate key immune cytokines like interferon−gamma, thereby shaping an immunosuppressive or immunostimulatory microenvironment.

Although this study provides mechanistic and clinical evidence, several limitations remain. First, the model’s predictive power is restricted in KRAS-mutant LUAD. KRAS activation simultaneously drives PI3K-AKT and MEK-ERK signalling (41) and enforces an immune landscape that is “hot yet exhausted”: PTEN is transcriptionally repressed, prompting compensatory TSPAN32 up-regulation that only partially restrains AKT, while MEK-ERK-mediated mTORC1 activity persists, preserving proliferation and metabolic immunosuppression. Consequently, despite increased CD8^+^ infiltration, elevated PD-L1 expression, expanded Tregs and T-cell exhaustion blunt the benefit of TSPAN32-driven PTEN restoration. Future models must incorporate additional factors to achieve accurate KRAS-mutant stratification. Second, the clinical datasets lacked details on radiotherapy regimens (fractionation, target volumes). Because OS and PFS were used as primary endpoints, variations in dose-fractionation could confound the relation between genetic signatures and survival. Prospective collections of dosimetric parameters are needed to validate the model and to test whether TSPAN32 expression interacts with specific schedules (e.g., hypofractionation vs conventional fractionation) before the signature can guide individualized radiotherapy. Finally, the specific mechanisms by which the TSPAN32-PTEN axis regulates radio-immunological responses and IL-17 signaling remain unclear, and its PTEN-dependency as well as the improvement of immunotherapy response require further validation through rescue experiments and combination studies with immune checkpoint inhibitors. Additionally, the direct causal relationship between TSPAN32 and IL-17 expression awaits further investigation. In summary, we constructed a robust radiotherapy-sensitivity risk model and identified TSPAN32 as a potential biomarker for selecting LUAD patients likely to benefit from radio-immunotherapy. Mechanistic studies suggest that TSPAN32 may regulate the immune microenvironment and enhance radiosensitivity through the PTEN-AKT- FOXO1 axis. Although its impact on immunotherapy response and detailed mechanisms require further exploration, future systematic studies of TSPAN32 are expected to guide the development of combined radio-immunotherapy strategies.

## Data Availability

The original contributions presented in the study are included in the article/**Supplementary Material**. Further inquiries can be directed to the corresponding authors.
