# Supplementary material for: TSPAN32 as a biomarker associated with radiotherapy and immune microenvironment remodeling in lung adenocarcinoma
Source: Front Oncol. 2026 Apr 14;16:1724489. doi: 10.3389/fonc.2026.1724489 (PMC13121124; doi:10.3389/fonc.2026.1724489)

Figure S1 The expression differences of the TSPAN32 gene across 31 types of tumors


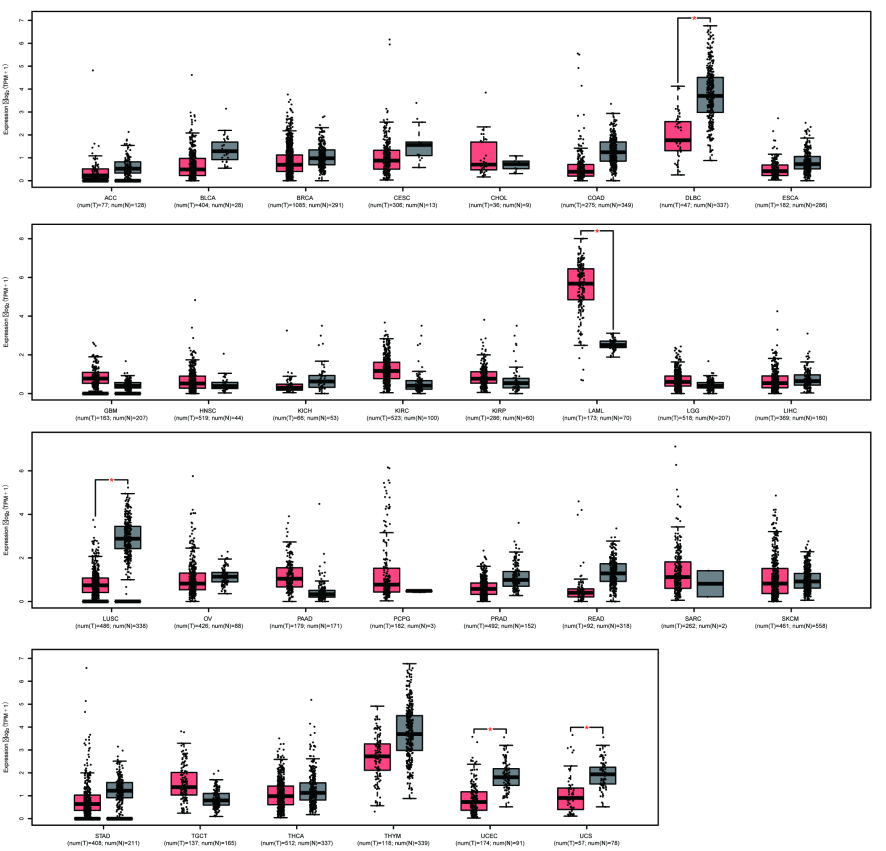


Figure S2 TSPAN32 expression and prognosis in EGFR- and KRAS-mutated lung adenocarcinoma subtypes

1. D. TSPAN32 expression in EGFR- and KRAS-mutated LUAD subtypes.
2. C. DFS and OS of TSPAN32 in EGFR-mutated LUAD
3. F. DFS and OS of TSPAN32 in KRAS-mutated LUAD


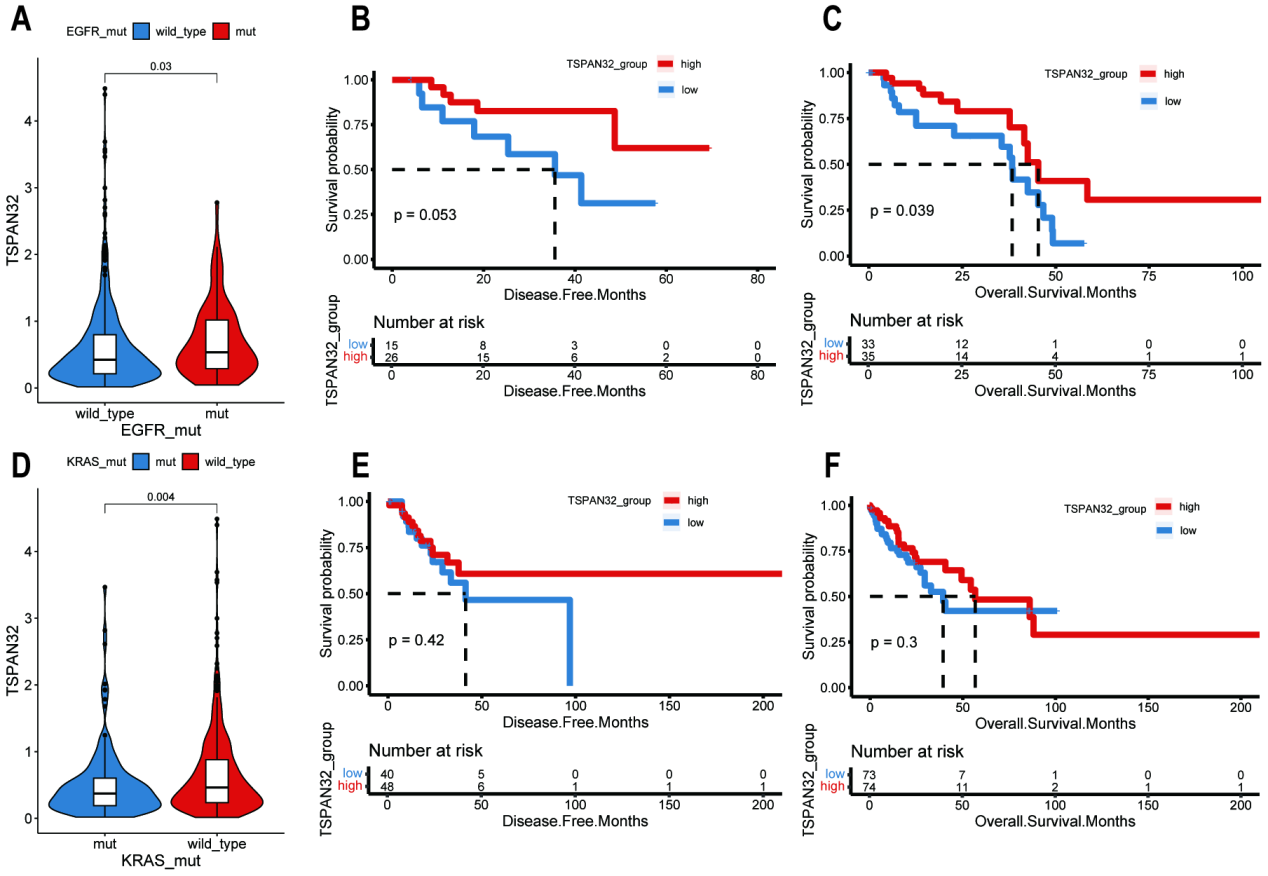


Figure S3 The stromal score status of the other 11 genes in the model besides TSPAN32


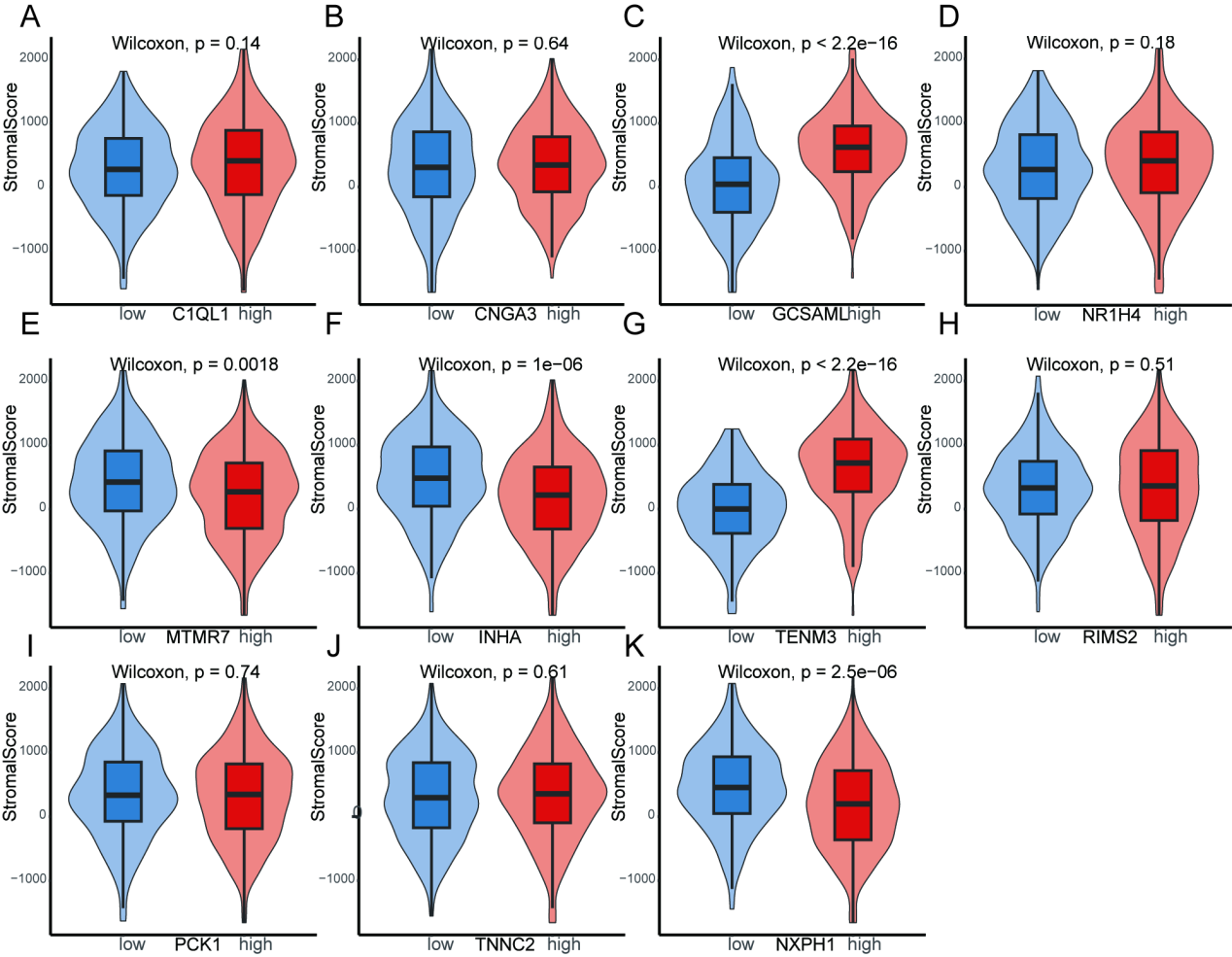


Figure S4 The immune score status of the other 11 genes in the model besides TSPAN32.


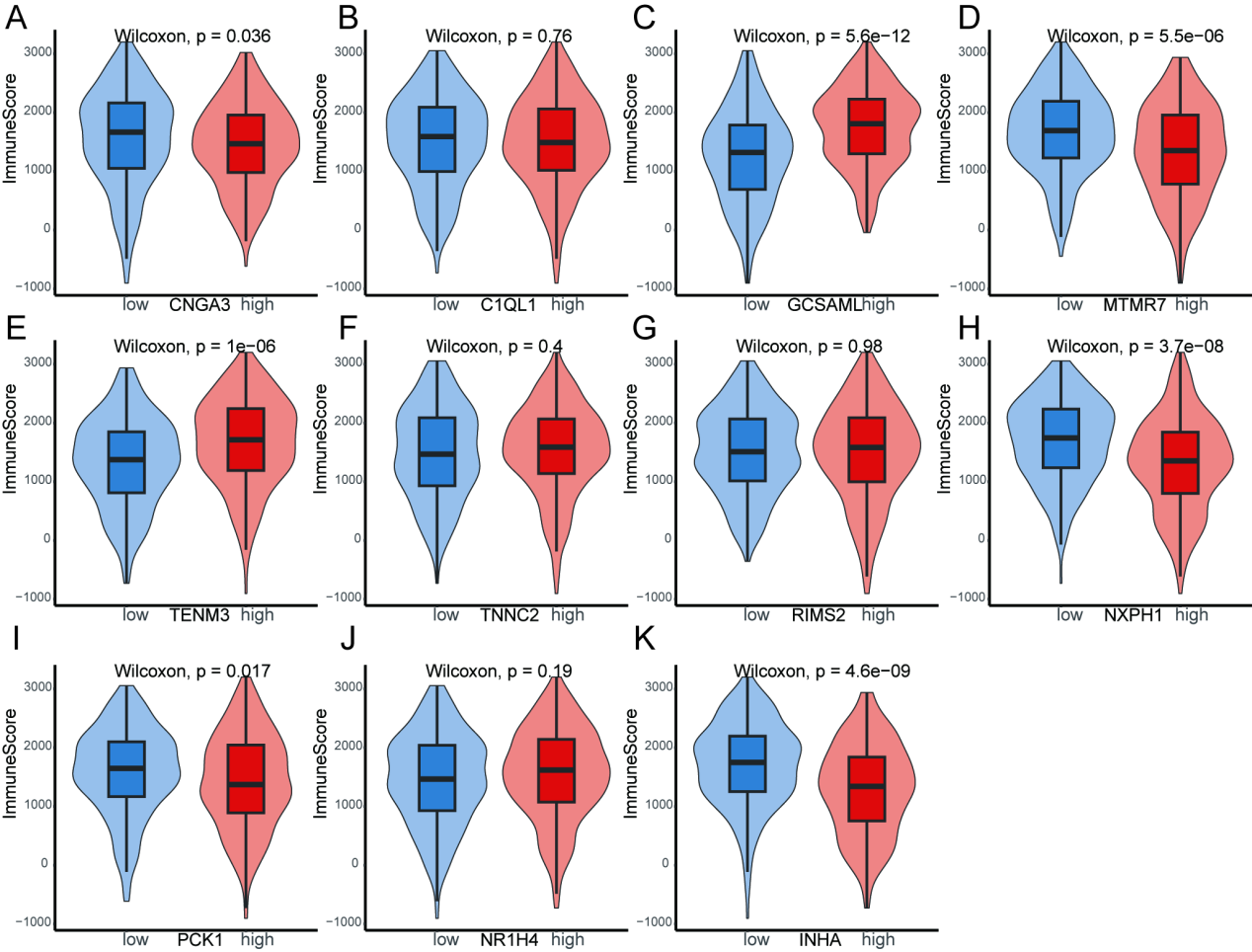


Figure S5 GO and KEGG analyses of the remaining 11 genes in the model excluding TSPAN32

A. GO analysis

B. KEGG analysis


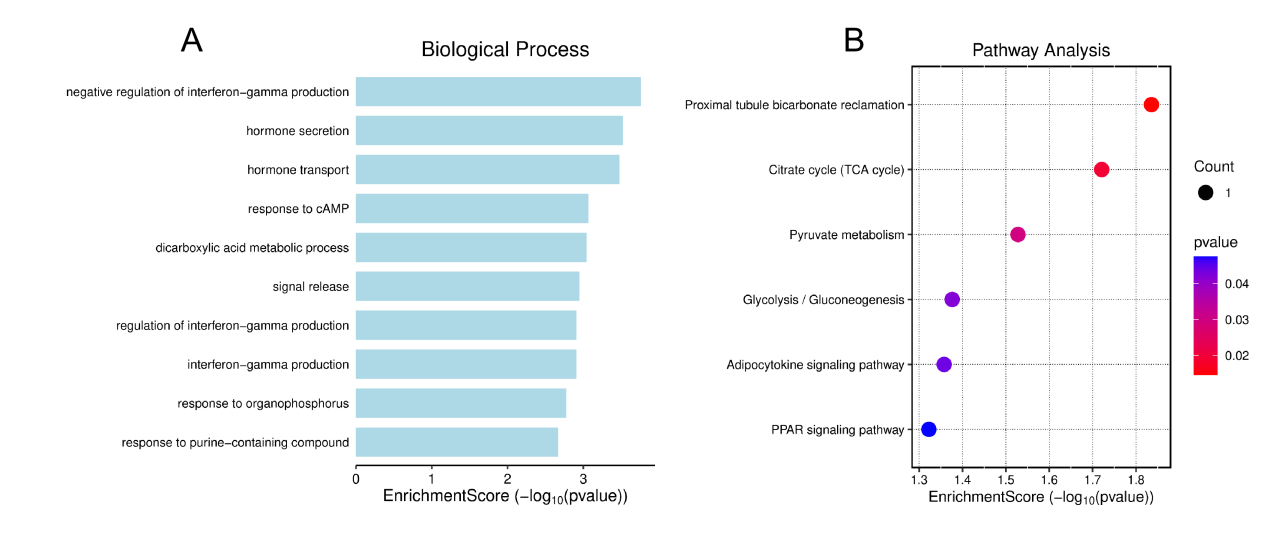


Figure S6The expression of TSPAN32 was detected by RT-qPCR and Western blotting (WB) in various non-small cell lung cancer (NSCLC) cell lines.

A. The expression of TSPAN32 was detected by RT-qPCR in BEAS-2B, A549, H1299, H1975, and H460 cells.

B. The expression of TSPAN32 was detected by WB in BEAS-2B, A549, H1299, H1975, and H460 cells. The error bars are absent as the results of only a single experiment were quantified here.


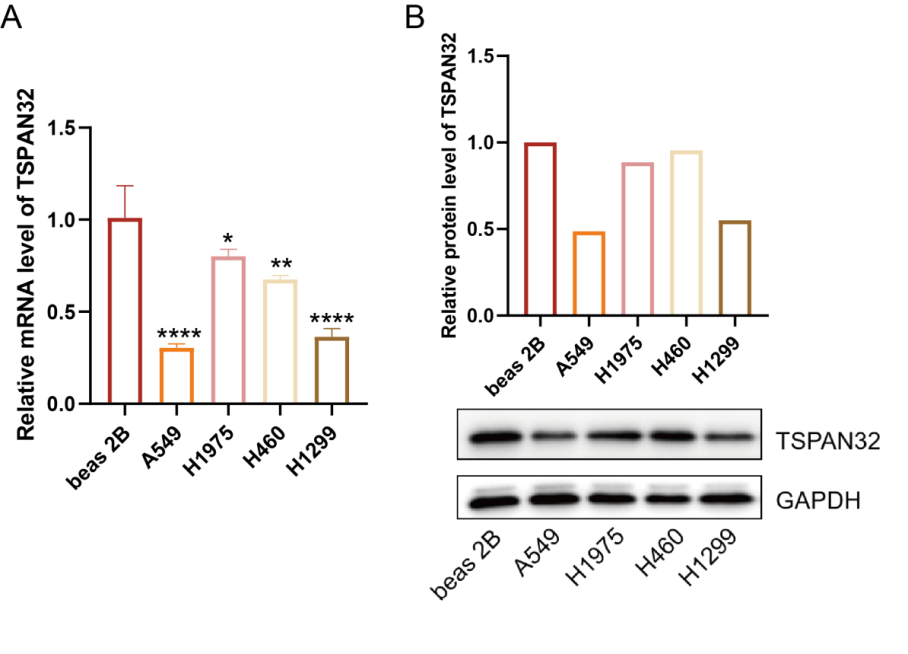

Supplement: Supplementary file 1 [file DataSheet1.docx]
